# PRDM1+ Malignant Cells Mediate an Immunosuppressive Landscape and Resistance to Neoadjuvant Chemoradiotherapy and Immunotherapy in Esophageal Squamous Cell Carcinoma

**DOI:** 10.1002/advs.202515207

**Published:** 2026-01-20

**Authors:** Dijian Shen, Rui Li, Yong She, Xuefei Liu, Yuanyang Huang, Yongling Ji, Keying Chen, Zhengbo Song, Runzhe Chen, Xuan Li, Qi Zhao, Qixun Chen, Ming Chen

**Affiliations:** ^1^ Department of Thoracic Oncology Surgery Hangzhou Institute of Medicine Zhejiang Cancer Hospital Chinese Academy of Sciences Hangzhou Zhejiang China; ^2^ Department of Radiation Oncology State Key Laboratory of Oncology in South China Guangdong Provincial Clinical Research Center for Cancer Sun Yat‐Sen University Cancer Center Guangzhou Guangdong China; ^3^ United Laboratory of Frontier Radiotherapy Technology of Sun Yat‐Sen University and Chinese Academy of Sciences Ion Medical Technology Co., Ltd. Guangzhou Guangdong China; ^4^ Department of Experimental Research State Key Laboratory of Oncology in South China Guangdong Provincial Clinical Research Center for Cancer Sun Yat‐sen University Cancer Center Guangzhou Guangdong China; ^5^ Department of Biochemistry School of Medicine Shenzhen Children's Hospital Southern University of Science and Technology Shenzhen Guangdong China; ^6^ Department of Radiation Oncology Hangzhou Institute of Medicine Zhejiang Cancer Hospital Chinese Academy of Sciences Hangzhou Zhejiang China; ^7^ Department of Population Health Sciences Duke University School of Medicine Durham North Carolina America; ^8^ Department of Phase I Clinical Trial Hangzhou Institute of Medicine Zhejiang Cancer Hospital Chinese Academy of Sciences Hangzhou Zhejiang China

**Keywords:** Esophageal squamous cell carcinoma (ESCC), Immune suppression, Lipid peroxidation, neoadjuvant immunotherapy and chemoradiotherapy (nICRT), PRDM1, Therapeutic resistance

## Abstract

The mechanisms underlying resistance to neoadjuvant immunotherapy and chemoradiotherapy (nICRT) in locally advanced esophageal squamous cell carcinoma (ESCC) remain poorly understood. Through a single‐arm phase II trial (*n* = 22) with 44.4‐month median follow‐up, we observed a significant survival disparity: patients achieving major pathologic response (MPR) exhibited superior 3‐year event‐free survival (EFS) and overall survival (OS), with no recurrence in MPR patients versus 71.4% recurrence in non‐major pathological response (NMPR) patients (HR = 17.69, 95% CI 2.25–139.20, *p* = 0.0063). Integrating single‐cell RNA/TCR sequencing and functional validation, we identified a PRDM1+ malignant cell subcluster enriched in NMPR patients and associated with treatment resistance. These cells exhibit strong lipid peroxidation characteristics, a state linked to the transcriptional activation of CTSB and MFSD12 mediated by PRDM1. This state renders the PRDM1+ malignant cell cluster more susceptible to ferroptosis induction. PRDM1+ cells further recruited immunosuppressive regulatory T cells (Tregs) through IL1A‐IL1R2 interactions and activated lipid‐metabolizing TREM2+ macrophages via CD47‐SIRPA signaling, fostering an immune‐evasive microenvironment. Conversely, MPR patients displayed expanded cytotoxic T‐effector clones with enhanced tumor‐killing capacity. Our findings identify PRDM1 as a key factor associated with nICRT resistance and suggest that targeting ferroptosis pathways or disrupting PRDM1+ cell‐mediated immune suppression may represent a viable strategy in ESCC. Clinical trial registration number: NCT03940001.

## Graphical abstract

Integrated scRNA‐seq, scTCR‐seq analysis, and functional assays identify PRDM1+ malignant epithelial cells with hyper lipid peroxidation characteristics that demonstrate reduced responsiveness to the nICRT treatment. Principal factor PRDM1 activates cysteine metabolism genes to modulate lipid peroxidation (an intrinsic cellular pathway related to ferroptosis), and simultaneously controls the expression of key ligands to remodel the immunosuppressive microenvironment (an extrinsic pathway involving Tregs and LAMs).

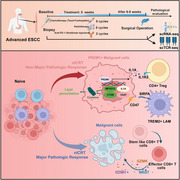

## 1 Introduction

Esophageal cancer ranks as the seventh most common cancer and the sixth leading cause of cancer‐related deaths on a global scale [1]. Esophageal squamous cell carcinoma (ESCC), the predominant histological subtype, is especially prevalent in Eastern Europe and Asia [2]. Currently, despite advancements in diagnosis and treatment, the prognosis of ESCC patients remains poor, with a 5‐year survival rate of 10%–30% [3, 4, 5]. Approximately 50% of esophageal cancer patients present with locally advanced disease and are eligible for potentially curative treatment at diagnosis [6]. Currently, based on the results of the CROSS and NEOCRTEC5010 trials [7, 8], neoadjuvant chemoradiotherapy (nCRT) plus esophagectomy is recommended as the standard treatment for locally advanced, operable ESCC [9]. Nevertheless, given the high rate of local or distant recurrence, the long‐term survival following the treatment approach of nCRT plus esophagectomy for ESCC is still far from satisfactory [8, 10]. Therefore, the establishment of novel and efficacious treatment strategies is of paramount importance for the further enhancement of the long‐term survival of ESCC patients.

The emergence of immunotherapy has brought new opportunities to improve the survival outcomes of esophageal cancer patients. For advanced‐metastatic esophageal cancer, immunotherapy, which is built on the success of immune checkpoint blockade (ICB), has obtained the approval of the U.S. Food and Drug Administration as the first and second‐line treatment modality [11, 12, 13, 14]. In addition, CheckMate‐577 has demonstrated that nivolumab nearly doubles the median disease‐free survival (DFS) to 22.4 months in the postoperative adjuvant treatment of esophageal cancer [15]. In the neoadjuvant treatment setting, nICRT and neoadjuvant immunochemotherapy (nICT) have shown good efficacy in operable locally advanced ESCC, with a pathological complete response (pCR) rate of up to about 50% in some studies and without an increase in surgical complication rates [16, 17, 18, 19]. Our previous pooled analysis of two phase II studies revealed that the nICRT group had a higher tumor pCR rate compared with the nICT group. However, even with the nICRT regimen having a higher pCR rate, more than 40% of patients still fail to achieve satisfactory outcomes. Previous studies reported the impacts of nICT on tumor microenvironment (TME) remodeling [20, 21]. However, they did not integrate the dynamics of immune cell clonotypes or the mutual crosstalk among different cells. Even more importantly, the resistance mechanism of nICRT treatment in operable locally advanced ESCC remains unclear, which is important to search for potential molecular targets to reverse therapeutic resistance.

In this study, we aim to address these knowledge gaps by elucidating the epithelial‐immune interactions of locally advanced ESCC and identifying principal factors that could mediate nICRT treatment resistance. We identify PRDM1+ malignant epithelial cells with hyperlipid peroxidation characteristics that demonstrate reduced responsiveness to the nICRT. This unique subtype can recruit Treg and lipid‐associated macrophages (LAM) that promote immune resistance. Overall, our results reveal a promising biomarker to guide patient selection for ESCC nICRT.

## 2 Results

### 2.1 MPR Correlates With Long‐Term Survival Benefit in Locally Advanced ESCC After nICRT

**FIGURE 1:**
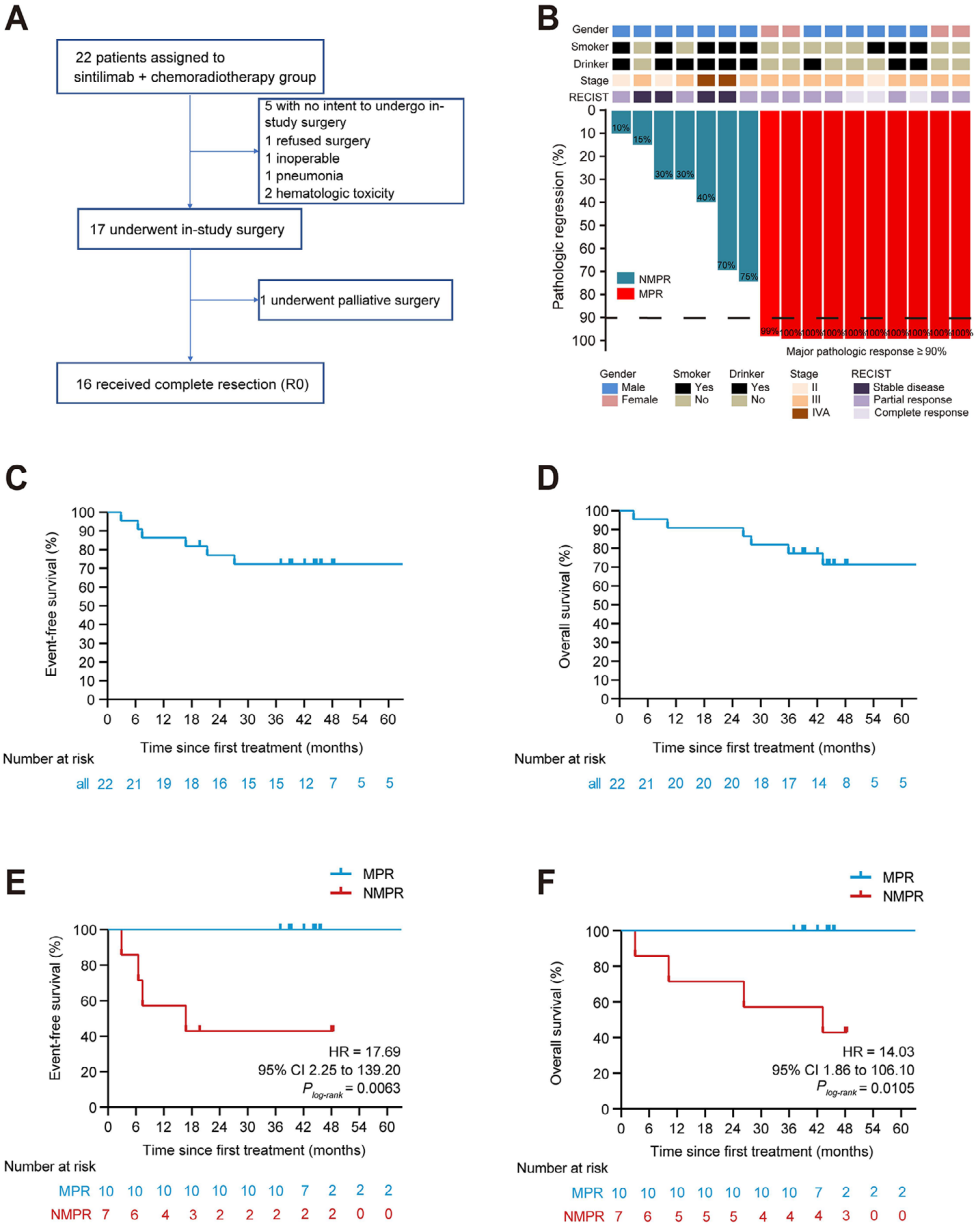
Clinicopathological characteristics and survival. (A) Patient disposition. (B) Clinical and pathological features are presented for each patient. RECIST, Response Evaluation Criteria in Solid Tumors (version 1.1). (C) Kaplan–Meier estimates of event‐free survival in the intention‐to‐treat population; Tick marks indicate censored data. (D) Kaplan–Meier estimates of overall survival in the intention‐to‐treat population; Tick marks indicate censored data. (E) Exploratory analysis of event‐free survival according to major pathological response. MPR is defined as no more than 10% viable tumor cells in the resected primary tumor; NMPR refers to cases with greater than 10% viable tumor cells. (F) Exploratory analysis of overall survival according to major pathological response to MPR.

Between May 2019 and March 2022, a total of 22 patients diagnosed with locally advanced ESCC were initially assessed for eligibility for this trial, of whom 17 received surgery (16 underwent R0 resection and one received palliative surgery) (Figure 1A). Patient characteristics are summarized in Table S1. All patients were Han Chinese. Median age was 64 years (range 47–75 years). 17 (77.3%) of 22 patients were male, and five (22.7%) were female. Most tumors were located in the distal third of the esophagus (12/22, 54.5%). Stage III ESCC accounted for 72.7% of these patients (16/22). As previously reported [22], the most common adverse events (AEs) among the 22 patients were lymphocytopenia (100.0%), anemia (95.5%), leukopenia (90.9%), esophagitis (68.2%), and decreased platelet count (63.6%). Most AEs were grade I or II, except for leukopenia. One patient developed grade 3 radiation pneumonitis. Seven patients (7/17, 41.2%) had pCR in both primary tumors and lymph nodes. MPR of primary tumors was observed in 10 patients (10/17, 58.8%). The median degree of pathologic regression of esophageal tumors was 100% (IQR 30%–100%), indicating substantial tumor response to nICRT (Figure 1B). Thirty‐day mortality was only observed in 1 (5.9%) patient due to postoperative anastomotic leakage and pulmonary infection, which eventually led to death from septic shock on the ninth postoperative day.

The median time from initial treatment to data cutoff on March 27, 2025, was 44.4 months (IQR 36.752.7). In the Full Analysis Set (FAS), the EFS rates were 86.4% (95%CI 73.2%–100.0%) at 12 months and 68.2% (95%CI 51.3%–90.7%) at 36 months (Figure 1C). OS outcomes are shown in Figure 1D. In FAS, the estimated 12‐ and 36‐month OS rates were 90.9% (95%CI 79.7%–100.0%) and 77.3% (95%CI 61.6%–96.9%), respectively. Median EFS and OS were not reached during the follow‐up period. Regarding the effect of tumor response on long‐term survival as a post‐hoc analysis, patients achieving an MPR had showed numerically improved OS (HR = 14.03, 95% CI 1.86–106.10, *p* < 0.0105) and EFS (HR = 17.69, 95% CI 2.25–139.20, *p* < 0.0063) (Figure 1E,F). Among the 10 patients who achieved MPR postoperatively, none experienced recurrence or metastasis within a three‐year period. Excluding one patient who died nine days after surgery, 4 out of the 6 patients with partial response (PR) or stable disease (SD) experienced tumor recurrence or metastasis within three years postoperatively.

### 2.2 Single‐Cell Transcriptomic Landscape in Locally Advanced ESCC

**FIGURE 2:**
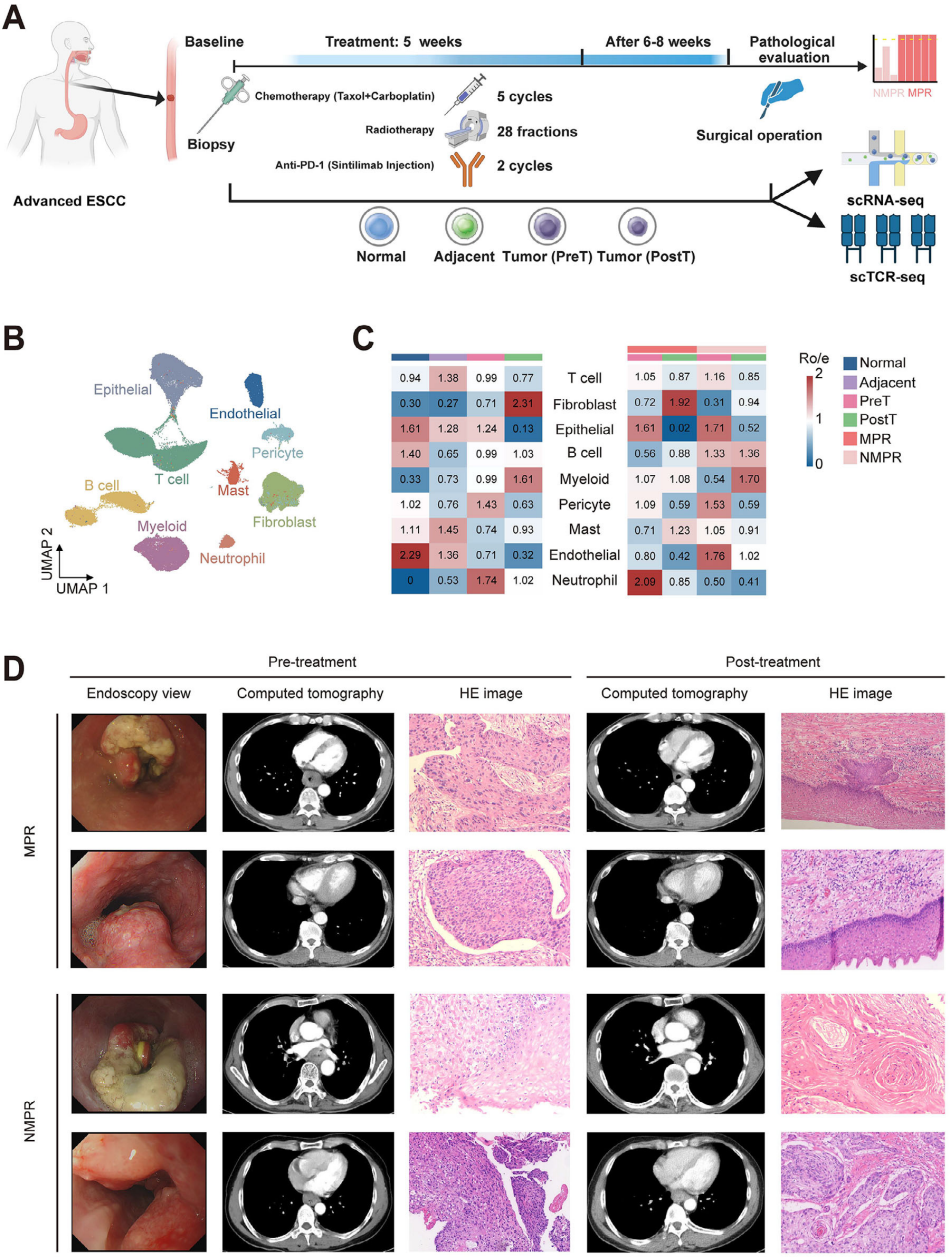
Single‐cell transcriptome profiling of locally advanced ESCC who received nICRT therapy. (A) The schematics of sample collection, scRNA‐seq, and scTCR‐seq analysis of ESCC. Normal, normal tissue; Adjacent, adjacent non‐cancerous tissue; PreT, pre‐treatment tumor tissue; PostT, post‐treatment tumor tissue. (B) The UMAP visualization is being performed on 120 665 cells from 26 samples, resulting in the identification of 9 major cell clusters. (C) The distribution preferences of major cell subclusters across different tissues and therapeutic response are estimated by Ro/e values. A larger Ro/e value indicates a higher degree of enrichment. (D) Representative images of MPR and NMPR patients before and after nICRT treatment are shown.

Our phase II clinical trial showed that more than 40% of patients still had poor efficacy. To explore the resistance mechanism to nICRT treatment in the resectable locally advanced ESCC, we obtained 26 samples from 9 patients to perform single‐cell RNA sequencing (scRNA‐seq) and single‐cell T‐cell receptor sequencing (scTCR‐seq) based on the above clinical trial. The samples included 4 normal tissues, 5 adjacent non‐cancerous tissues, 9 pre‐treatment tumor biopsies, and 8 post‐treatment tumor surgical samples (Patient 4's postoperative pathological specimen failed to undergo sequencing due to contamination, Figure 2A). These ESCC patients were categorized into two groups: MPR (*n* = 5) and NMPR (*n* = 4), based on whether the estimated percentage of residual tumor cells was less than 10%. The clinical and pathological features of 9 ESCC patients are described in Table S2. After removing doublets and quality‐control filtering, we obtained 120,665 high‐quality cells, which were classified into 9 major cell types according to corresponding marker genes (Figure S2B; Figure S1A–C), including B cell, T cell, myeloid, mast cell, epithelial cell, fibroblast, pericyte, endothelial cell, and neutrophil. The distribution of cell types across different groups or tissues was shown in Figure S2B and Figure S1D. After nICRT treatment, there was a significant increase in fibroblasts, while there was a notable decrease in epithelial cells. Consistent with tumor pathological assessment, scRNA‐seq analysis showed that some epithelial cells remained in NMPR patients, indicating nICRT therapy does not appear to work as well in these patients (Figure 2C). Representative radiological, endoscopic, and histopathological images of tumors from MPR and NMPR patients are shown (Figure 2D).

### 2.3 PRDM1+ Epithelial Cell Elevated Lipid Peroxidation and Correlated with Resistance to nICRT Therapy

**FIGURE 3:**
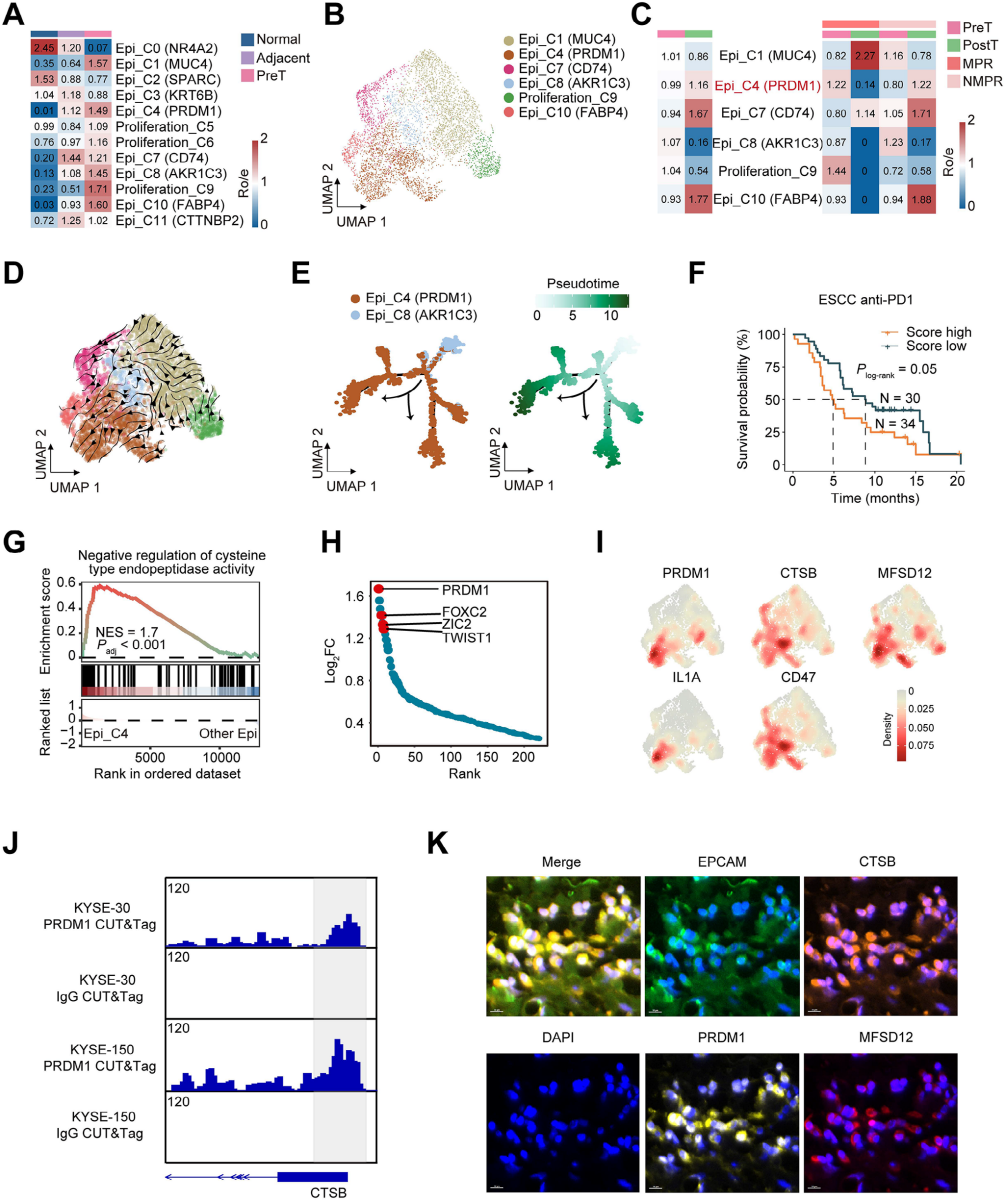
Epithelial cell clusters with elevated lipid peroxidation and PRDM1 expression were enriched in NMPR patients. (A) Tissue distribution of different epithelial cells estimated by Ro/e score. (B) UMAP showing 6 malignant cell subtypes. (C) Tissue or efficacy distribution of different malignant cells estimated by Ro/e score. (D) UMAP of RNA velocity of 6 malignant cell subtypes by scvelo. (E) Pseudotime‐ordered analysis of 2 malignant cell subtypes (Epi_C4 and Epi_C8) inferred by Monocle 2. (F) Kaplan–Meier estimation of overall survival in ESCC patients based on Epi_C4 signature score in a PD‐1 inhibitor (sintilimab) treatment cohort. (G) GSEA showed negative regulation of the cysteine protease pathway in PRDM1+ malignant cells. The NES and FDR are included. (H) Differential expressed transcription factors between PRDM1+ malignant cells and the other cell subtypes. *Y*‐axis indicates log_2_(fold change), genes ordered by log_2_(fold change) along the *x*‐axis. (I) Density of *PRDM1*, *CTSB*, *MFSD12*, *IL1A* and *CD47* expression of all malignant cells. (J) Genome browser tracks of PRDM1 CUT&Tag‐seq at the CTSB genomic locus in ESCC cells. (K) Representative mIHC staining of an NMPR ESCC tumor sample after treatment, with markers including EPCAM, PRDM1, CTSB, MFSD12, and DAPI. Scale bar:10 µm.

We subclassified the epithelial cells expressing *EPCAM* and *KRT5*, and found 12 cell subtypes that were present in all patients (Figure S2A). The marker genes of epithelial cell subtypes are shown in Figure S2B. We inferred that six of the twelve subpopulations were malignant cell subtypes based on their enrichment in different tissues and the results of inferCNV (Figure 3A,B; Figure S2C). Among 6 malignant cell subtypes, Epi_C4 (PRDM1) highly expressing *LAMC2*, *CCL20*, and *PRDM1*, showed an increase in NMPR patients after nICRT treatment, while Epi_C8 (AKR1C3) expressing *AKR1C3* and *WIF1* exhibited a marked decrease (Figure 3C; Figure S2D). We predicted potential differentiation directions of 6 malignant cell subtypes by two methods, and results suggested that the flow directions were from Epi_C8 (AKR1C3) to Epi_C4 (PRDM1) (Figure 3D,E). Moreover, we found that Epi_C4 (PRDM1) was enriched in pathways related to epithelial‐mesenchymal transition, hypoxia, collagen formation, and extracellular matrix organization (Figure S2E), which were commonly associated with tumor occurrence, metastasis, and treatment resistance [23, 24]. Some ESCC bulk RNA‐seq results showed that the Epi_C4 signature score was significantly higher in tumor tissue than in normal tissue (Figure S2F). Furthermore, analysis of a PD‐1 inhibitor (sintilimab) treatment ESCC cohort found that patients with high Epi_C4 signature scores had shorter survival times compared to those with lower scores (Figure 3F).

**FIGURE 4:**
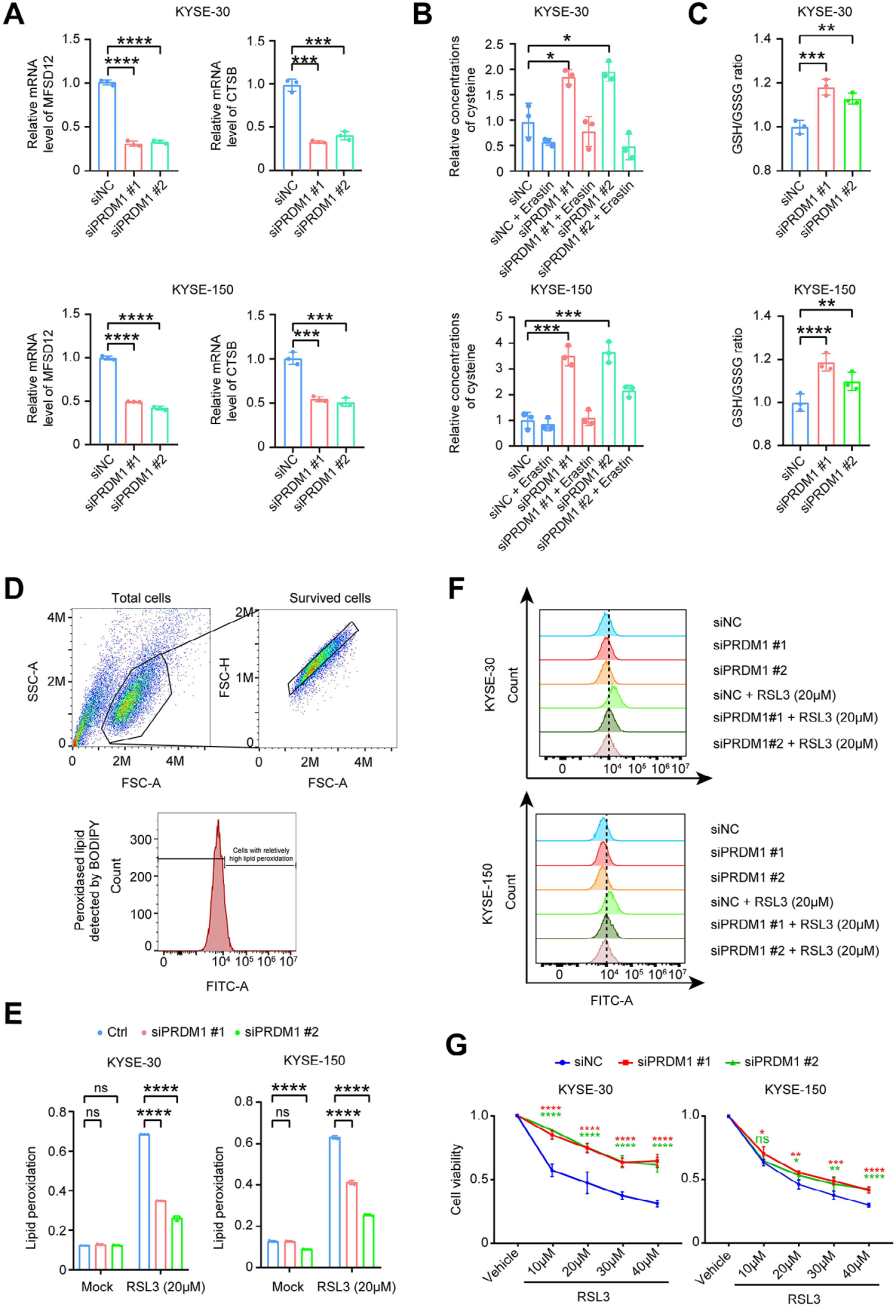
A PRDM1‐positive cell subpopulation critically drives lipid peroxidation accumulation through direct transcriptional regulation of cysteine metabolism. (A) Relative mRNA levels of MFSD12 and CTSB in KYSE‐30 and KYSE‐150 cells transfected with PRDM1‐targeting siRNA (siPRDM1) or non‐targeting control siRNA (siNC), as quantified by qRT‐PCR. (B) Relative cysteine concentration in cellular proteins was measured by colorimetric assay in untreated cells or cells exposed to 20 µm Erastin for 24 h. (C) Ratio of reduced glutathione (GSH) to oxidized glutathione (GSSG) was determined by colorimetric assay in siRNA‐transfected cells. (D,E) Percentage of cells with elevated lipid peroxidation after 24 h treatment with 20 µm RSL3 or vehicle control (DMSO, Mock), determined by flow cytometry using the lipid peroxidation sensor BODIPY 581/591 C11 (higher FITC‐A channel intensity indicates increased oxidized lipids). (F) Representative flow cytometry histograms of BODIPY staining in RSL3‐treated versus Mock‐treated cells. (G) Cell viability was assessed by MTT assay following treatment with DMSO (1:250 dilution) or four graded concentrations of RSL3. Data are presented as mean ± standard error of the mean (SEM) from three independent experiments, ns denotes not significant; ^*^ *p* < 0.05, ^**^ *p* < 0.01, ^***^ *p* < 0.001, ^****^ *p* < 0.0001 (analyzed by one‐way ANOVA).

Gene set enrichment analysis (GSEA) revealed downregulation of cysteine‐type endopeptidase activity in Epi_C4 cells (Figure 3G). PR domain zinc finger protein 1 (*PRDM1*) as a transcription factor was significantly upregulated in the Epi_C4 cells (Figure 3H). Compared to other malignant cell subtypes, Epi_C4 (PRDM1) specifically overexpressed *PRDM1*, *IL1A*, *CD47*, and some lipid peroxidation‐associated genes, including *CTSB* [25] and *MFSD12* [26] (Figure 3I). PRDM1 Cleavage Under Targets and Tagmentation sequencing (CUT&Tag‐seq) demonstrated strong binding of PRDM1 to promoter regions of CTSB and MFSD12 in two ESCC (KYSE‐30 and KYSE‐150) cell lines, suggesting its potential role in facilitating their transcription (Figure 3J; Figure S2G). Multiplex immunohistochemistry (mIHC) experiments found that an enhanced lipid peroxidation characteristic epithelial cell subcluster with high protein CTSB and MFSD12 indeed existed in NMPR patients (Figure 3K; Figure S2H). To validate the results from our CUT&Tag‐seq and mIHC experiments, we performed *PRDM1* depletion in esophageal cancer cells. Quantitative real‐time PCR (RT‐qPCR) analysis demonstrated significant reductions in messenger RNA (mRNA) levels of both MFSD12 and CTSB (Figure 4A; Figure S3A). As key regulators of cellular cysteine metabolism, PRDM1 was associated with an upregulation in total cysteine concentration (Figure 4B) and an increased GSH/GSSG ratio (Figure 4C), collectively indicating enhanced cellular detoxification capacity against lipid peroxidation. Conversely, when PRDM1 was overexpressed, the mRNA levels of MFSD12 and CTSB significantly increased, accompanied by decreased intracellular cysteine concentrations and a decreased GSH/GSSG ratio (Figure S3B–E). The results suggest that lipid peroxidation is prone to accumulate due to impaired clearance capacity. Flow cytometry (Figure 4D) demonstrated that *PRDM1* depletion did not significantly alter basal lipid peroxidation levels but substantially attenuated RSL3 (a ferroptosis inducer)‐triggered lipid peroxidation (Figure 4E,F). Conversely, *PRDM1* overexpression exacerbated lipid peroxidation (Figure S3F,G). The intracellular accumulation of lipid peroxidation may drive tumor progression and therapeutic resistance through multiple pathways, including epigenetic reprogramming, modulation of membrane repair enzyme systems, and remodeling of the TME. In this study, we hypothesize that the PRDM1‐high malignant cells mediate resistance to nICRT in esophageal carcinoma via the induction of lipid peroxidation.

Ferroptosis, a recently identified non‐apoptotic cell death modality mechanistically dependent on lipid peroxidation, is particularly relevant given that PRDM1‐high cells drive upregulation of lipid peroxidation [27]. This raises a critical therapeutic hypothesis: Could pharmacological induction of ferroptosis selectively eliminate these hyper‐peroxidized cells to reverse therapy resistance? MTT assays demonstrated that while PRDM1 depletion attenuated RSL3‐induced cell death (Figure 4G), its overexpression conversely promoted cell death in both KYSE‐30 and KYSE‐150 cells (Figure S3F,G). Notably, this pro‐death effect was significantly reversed by co‐treatment with the ferroptosis inhibitor ferrostatin‐1 (Fer‐1) or the cysteine supplement N‐acetylcysteine (Figure S3H,I).

Collectively, we have identified a PRDM1‐positive cell subpopulation associated with lipid peroxidation accumulation through direct transcriptional regulation of cysteine metabolism, which may represent a key mechanism underlying resistance to nICRT in esophageal carcinoma. Furthermore, our functional studies demonstrate that pharmacological induction of ferroptosis selectively eliminates PRDM1‐high cells, suggesting that targeting this pathway could be a promising strategy to reverse therapeutic resistance in this context. To further strictly delineate the regulatory hierarchy and rule out the possibility that the metabolic state secondarily dictates the immune phenotype, we investigated whether manipulating ferroptosis status influences the expression of immune ligands. qPCR analysis revealed that pharmacological perturbation of ferroptosis, using either the inducer RSL3 or the inhibitor Fer‐1, did not alter the mRNA expression of CD47 or IL1A in KYSE‐30 and KYSE‐150 cells (Figure S4A). This finding reinforces the hypothesis that PRDM1 regulates intrinsic metabolic vulnerability (lipid peroxidation) and extrinsic immunosuppressive signaling via distinct, parallel mechanisms rather than through a linear cascade interaction.

### 2.4 PRDM1+ Malignant Cells and LAMs Crosstalk Mediate nICRT Resistance by CD47–SIRPA Axis

**FIGURE 5:**
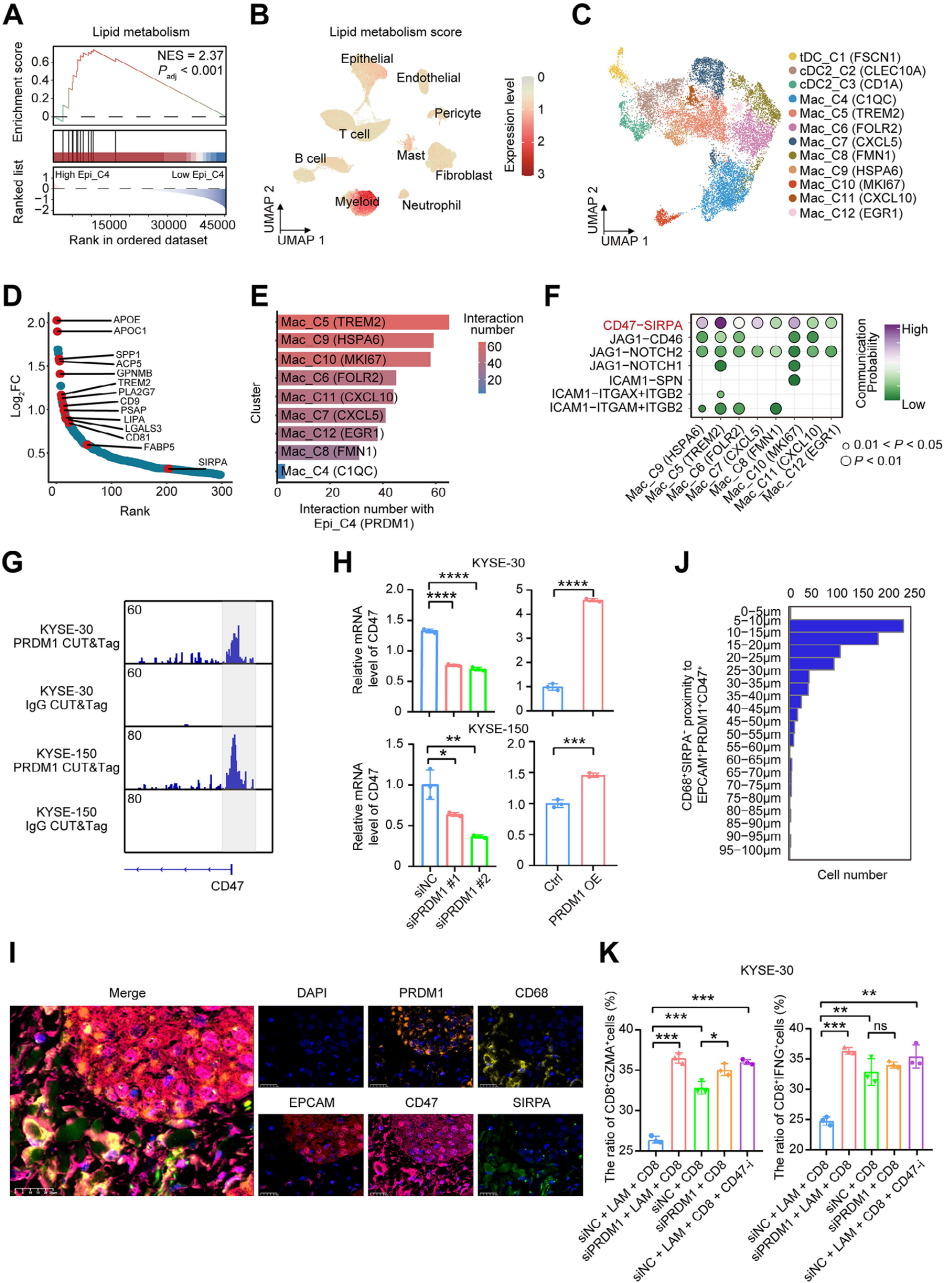
Cell communication of PRDM1+ malignant cells with LAMs in NMPR patients. (A) GSEA showing lipid metabolism pathway was upregulated in ESCC patients with a higher Epi_C4 signature score. patients were divided into high and low groups based on the Epi_C4 signature score in a published ESCC cohort. The NES and FDR are included. (B) UMAP plots presenting lipid metabolism signature score in all cells. (C) UMAP showing 12 myeloid cell subtypes. (D) Differential expressed genes between Mac_C5 cells and the other cell subtypes. The *Y*‐axis indicates log_2_(fold change), with genes ordered by log_2_(fold change) along the *x*‐axis. (E) Number of significant ligand‐receptor pairs between PRDM1+ malignant cells and macrophage cell subtypes. (F) Dotplot showing the indicated ligand‐receptor pairs between PRDM1+ malignant cells and macrophage cell subpopulations in the tumor. *p*‐values were determined using a one‐sided permutation test. (G) Genome browser tracks of PRDM1 CUT&Tag‐seq at the CD47 genomic locus in ESCC cells. (H) Effect of PRDM1 depletion (siPRDM1) and overexpression on CD47 expression in ESCC cells. (I) mIHC staining of EPCAM, PRDM1, CD47, CD68, SIRPA, and DAPI in an NMPR ESCC tumor tissue sample after treatment. Scale bars: 25 µm. (J) The HALO software was used to quantify the distribution of CD68+SIRPA+ Macrophage cells surrounding EPCAM+PRDM1+CD47+ cancer cells in mIHC‐stained patient whole‐slide images. (K) Flow cytometry quantification of the proportion of GZMA+CD8+ T cells (left panel) and IFNG+CD8+ T cells (right panel). For (H,K), data are presented as mean ± SEM from three independent experiments; ns denotes not significant; ^*^ *p* < 0.05, ^**^ *p* < 0.01, ^***^ *p* < 0.001, ^****^ *p* < 0.0001 (analyzed by unpaired two‐tailed Student's *t*‐test and one‐way ANOVA).

We analyzed a published ESCC cohort and divided patients into high and low groups based on the Epi_C4 signature score. Patients with higher Epi_C4 signature scores showedsignificant activation of pathways related to lipid metabolism (Figure 5A). To our surprise, some of the myeloid cells had a higher lipid metabolism signature score compared to epithelial cells (Figure 5B). Next, we subclassified myeloid cells and identified 12 subtypes, including three dendritic cell (DC) subtypes and nine macrophage subtypes (Figure 5C). Subclusters expressing resident‐like markers, including *FOLR2*, *F13A1*, and *SELENOP*, were denoted as tissue‐resident macrophages (Mac_C6), and subclusters expressing *CXCL9*, *ISG15*, and *CXCL10* were defined as anti‐tumor macrophages [28] (Mac_C11), whereas clusters expressing *TREM2* and *SPP1* were marked as tumor‐associated macrophages (TAMs) [29, 30] (Mac_C5, Mac_C7). Consistent with the above, Mac_C7 exhibited the strongest angiogenesis phenotype, Mac_C6 demonstrated the highest phagocytosis score, and Mac_C5 showed the greatest lipid metabolism score (Figure S5A,B). Notably, Mac_C6 and Mac_C8 subclusters with robust phagocytic phenotypes were significantly increased after nICRT treatment, especially in MPR patients.

The Mac_C5 subcluster was mainly enriched in NMPR patients, and even after treatment, it still exhibited a high abundance in NMPR patients (Figure S5C). The Mac_C5 subcluster was defined as LAMs due to high expression of many lipid metabolism‐associated genes, including *APOE*, *APOC1*, *SPP1*, *TREM2*, and *LIPA* (Figure 5D). Similarly, we collected lipid metabolism‐associated gene sets from two studies [31, 32] and found a great overlap with genes uniquely highly expressed in the Mac_C5, confirming that cells in Mac_C5 are LAMs (Figure S5D). Analysis of bulk RNA‐seq cohorts showed significantly higher lipid metabolism scores in tumor versus normal tissues (Figure S5E). Furthermore, high lipid metabolism scores were correlated with shorter survival in TCGA‐ESCC and anti‐PD‐1‐treated ESCC cohorts (Figure S5F).

We speculated that Epi_C4 (PRDM1) cells might facilitate immune evasion by recruiting LAMs, and subsequently investigated the interactions between them. Cell communication analysis showed that Epi_C4 (PRDM1) had the strongest crosstalk with Mac_C5, especially through the ligand‐receptor interaction of CD47 with SIRPA (Figure 5E,F). We also detected a significantly positive correlation between the Epi_C4 signature score and lipid metabolism score by the RNA‐seq data in published ESCC databases (Figure S5G). Gene expression analysis showed that the lipid metabolism patterns in NMPR patients were more activated after nICRT treatment, specifically manifested by upregulation of lipid synthesis, transport, and degradation (Figure S6A,B). PRDM1 CUT&Tag‐seq and genetic manipulation experiments indicated that PRDM1 may act as a transcription factor for CD47 (Figure 5G,H). mIHC experiments and HALO image analysis demonstrated that CD47+PRDM1+ epithelial cells were closely connected with SIRPA+CD68+ macrophage cells (Figure 5I,J; Figure S6C,D).

To further investigate whether PRDM1+ cancer cells can induce an immunosuppressive microenvironment through LAMs, we established an indirect co‐culture system of cancer cells, CD8+ T cells, and LAMs (Figure S7A). We differentiated the human‐derived monocyte cell line THP‐1 into M0 and then induced them into LAM in vitro. Next, we used Nile red staining of the cells to demonstrate that the successfully induced LAM contained abundant lipid droplets (Figure S7B). CD8+ T cells were isolated from ESCC patients’ PBMCs. We observed that the proportion of cytotoxic CD8+ T cells (CD8+IFNG+ and CD8+GZMA+) was the lowest in the control group, which was as expected, as the cancer cells induced an immunosuppressive microenvironment through LAMs (Figure 5K; Figure S7C,D). When *PRDM1* depletion in the cancer cells or a CD47 inhibitor interfered, the proportion of cytotoxic CD8+ T cells significantly increased, which is conducive to anti‐tumor immunity (Figure 5K; Figure S7C,D). This highlights CD47 as a promising therapeutic target for locally advanced ESCC patients with immunotherapy resistance. We then calculated the interaction scores of CD47–SIRPA pairs by adjusting the corresponding cell fractions, and evaluated their relationships with patient survival time in a cohort of ESCC patients receiving PD‐1 therapy. The results showed that ESCC patients with high scores had shorter survival times than those with low scores (Figure S6E). Overall, these findings suggest that PRDM1+ malignant cells interact with LAMs through the CD47–SIRPA axis to enhance microenvironmental lipid metabolism, which may mediate nICRT treatment resistance.

### 2.5 PRDM1+ Malignant Cells Recruits Tregs via the IL1A‐IL1R2 Pair in NMPR Patients

**FIGURE 6:**
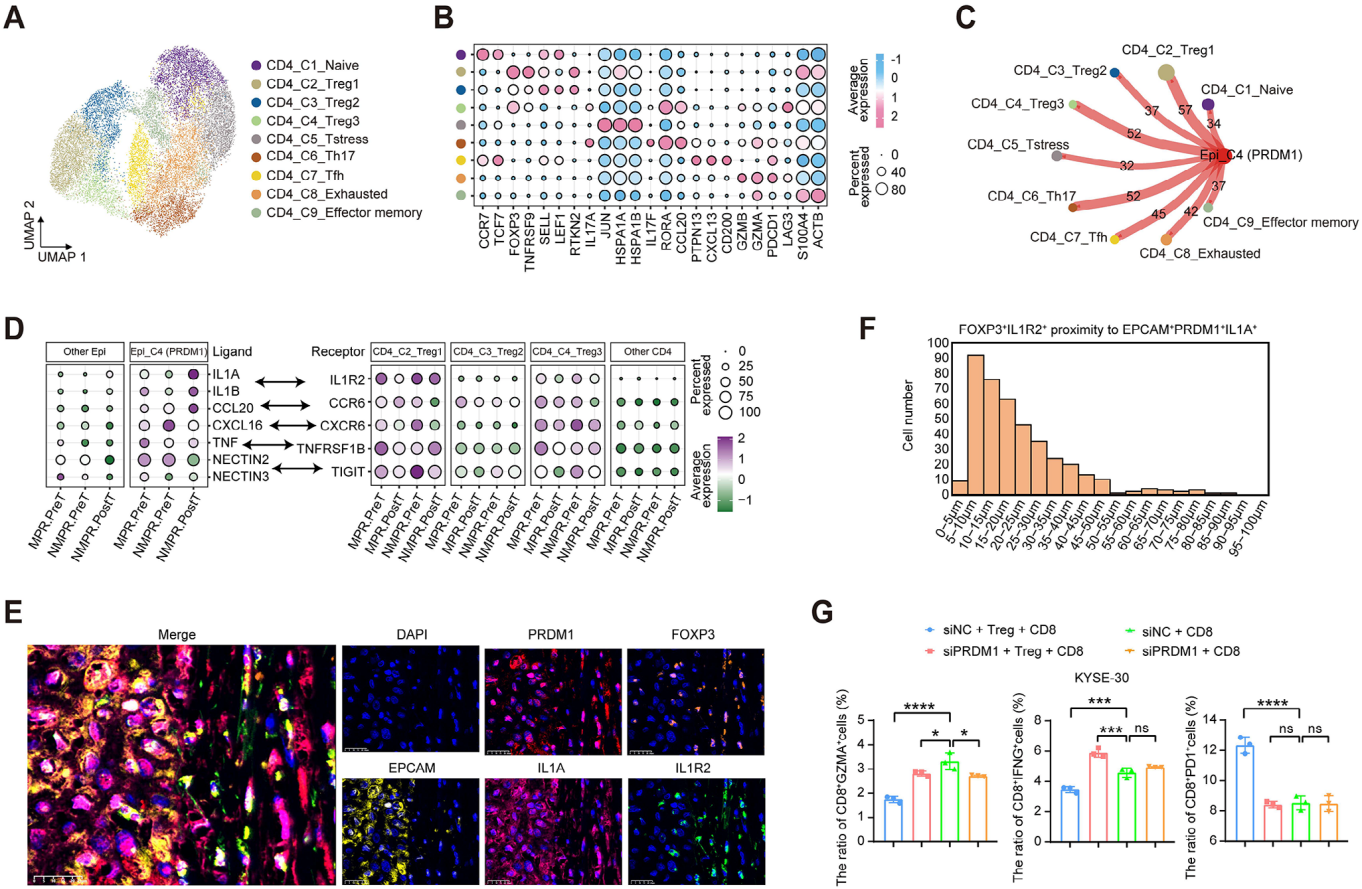
PRDM1+ malignant cells recruit immune‐suppressive Tregs via the IL1A‐IL1R2 pair in NMPR patients. (A) UMAP showing 9 CD4 cell subtypes. (B) Dotplot displaying the expression levels of the selected markers in different CD4 cell subtypes. Dot size reflects the fraction of expressing cells, and the colors denote normalized gene expression levels. (C) Number of significant ligand‐receptor pairs between PRDM1+ malignant cells and CD4 cell subtypes. (D) In the tumor samples of different groups, the expression levels of *IL1A*, *IL1B*, *CCL20*, *CXCL16*, *TNF*, *NECTIN2* and *NECTIN3* in different cancer cell subtypes (left panel). And the expression levels of *IL1R2*, *CCR6*, *CXCR6*, *TNFRSF1B*, and *TIGIT* in different CD4 cell subtypes (right panel). (E) Representative mIHC staining of NMPR ESCC tumor samples after treatment, with markers including: EPCAM, PRDM1, IL1A, FOXP3, IL1R2, and DAPI. Scale bars: 25 µm. (F) The HALO software was used to quantify the distribution of FOXP3+IL1R2+ Treg cells surrounding EPCAM+PRDM1+IL1A+ cancer cells in mIHC‐stained patient whole‐slide images, from close to distant. (G) Flow cytometry quantification of the proportions of GZMA+CD8+ T cells (left panel), IFNG+CD8+ T cells (middle panel), and PD1+CD8+ T cells (right panel). For (G), data are presented as mean ± SEM from three independent experiments; ns denotes not significant; ^*^ *p* < 0.05, ^**^ *p* < 0.01, ^***^ *p* < 0.001, ^****^ *p* < 0.0001 (analyzed by one‐way ANOVA).

We subclustered CD4+ T cells and obtained 9 subtypes according to the RNA level of canonical markers (Figure 6A,B), including naive (C1), three regulatory (C2, C3, C4), stress (C5), type 17 helper (C6), follicular helper (C7), exhausted (C8) and effector memory CD4+ T cells (C9). The Treg cells expressed some classical genes (*FOXP3*, *TNFRSF9*, *LAG3*, *CTLA4*, and *IKZF2*), which play an immunosuppressive role in the tumor‐immune microenvironment. We observed that the MPR patients experienced nearly complete elimination of Treg cells after nICRT treatment, whereas the NMPR patients exhibited a higher abundance of Tregs both before and after treatment (Figure S8A,B). We observed that metabolic features such as oxidative phosphorylation and glycolysis were highly active in the CD4_C2_Treg1 subpopulation (Figure S8B).

We investigated the crosstalk between PRDM1+ malignant cells and CD4+ T cells, and the results showed that Epi_C4 (PRDM1) cells harbored the highest number of ligand‐receptor interactions with CD4_C2_Treg1 (Figure 6C). Furthermore, we evaluated the correlation of ligand‐receptor pairs at the subcluster level and found that the ligand‐receptor pairs IL1A–IL1R2, NECTIN2–TIGIT, and CCL20–CCR6 were significantly enriched between Epi_C4 (PRDM1) cells and CD4_C2_Treg1 in NMPR patients (Figure 6D). Notably, a significant positive correlation between Epi_C4 (PRDM1) cells and CD4_C2_Treg1 was observed across multiple ESCC bulk RNA‐seq datasets (Figure S8C). In the PRDM1 CUT&Tag‐seq, we found that the transcription factor PRDM1 could bind to the promoter region of IL1A (Figure S8D). We subsequently performed genetic manipulation experiments in two ESCC cell lines to validate this, and the results indicated that the RNA level of IL1A was significantly reduced in *PRDM1* depletion cells, while in cells with *PRDM1* overexpression, the RNA levels of IL1A were significantly increased (Figure S8E,F). mIHC further showed spatial proximity between IL1A+PRDM1+ tumor cells and IL1R2+ Tregs, which was quantitatively confirmed by HALO image analysis (Figure 6E,F; Figure S8G,H), indicating a close physical interaction that may facilitate signaling via the IL1A–IL1R2 axis.

To further investigate whether PRDM1+ cancer cells can induce an immunosuppressive microenvironment through Tregs, we established an indirect co‐culture system of cancer cells, CD8+ T cells, and Tregs (Figure S9A). Following in vitro induction of Tregs from isolated CD4+ T cells derived from PBMCs, successful differentiation was confirmed by flow cytometry using the characteristic marker profile of CD4+ CD25+ CD127 low Tregs (Figure S9B). Control groups showed minimal cytotoxic CD8+ T cells and maximal exhausted CD8+ T cells. When PRDM1 was depleted in the cancer cell line, the proportion of cytotoxic CD8+ T cells significantly increased, and the proportion of exhausted CD8+ T cells significantly decreased (Figure 6G; Figure S9C,D). Importantly, in the presence of Tregs, compared with the siNC group, tumor cells with siPRDM1 consistently led to increased T cell effector function and decreased T cell exhaustion. In contrast, in the absence of Tregs, there were minimal changes in T cell effector function and exhaustion between the siPRDM1 group and the siNC group. This indicates that the immunosuppressive microenvironment was partially alleviated, which is conducive to anti‐tumor immunity and that the sustained presence of Tregs is more likely to be one of the causes of resistance. Collectively, these findings support the hypothesis that PRDM1+ malignant cells in NMPR patients may promote an immunosuppressive microenvironment, at least in part through recruitment of Tregs via IL1A–IL1R2 signaling, contributing to resistance to nICRT.

### 2.6 nICRT‐Related Changes in the CD8+ T Cell Infiltration and Cytotoxicity

**FIGURE 7:**
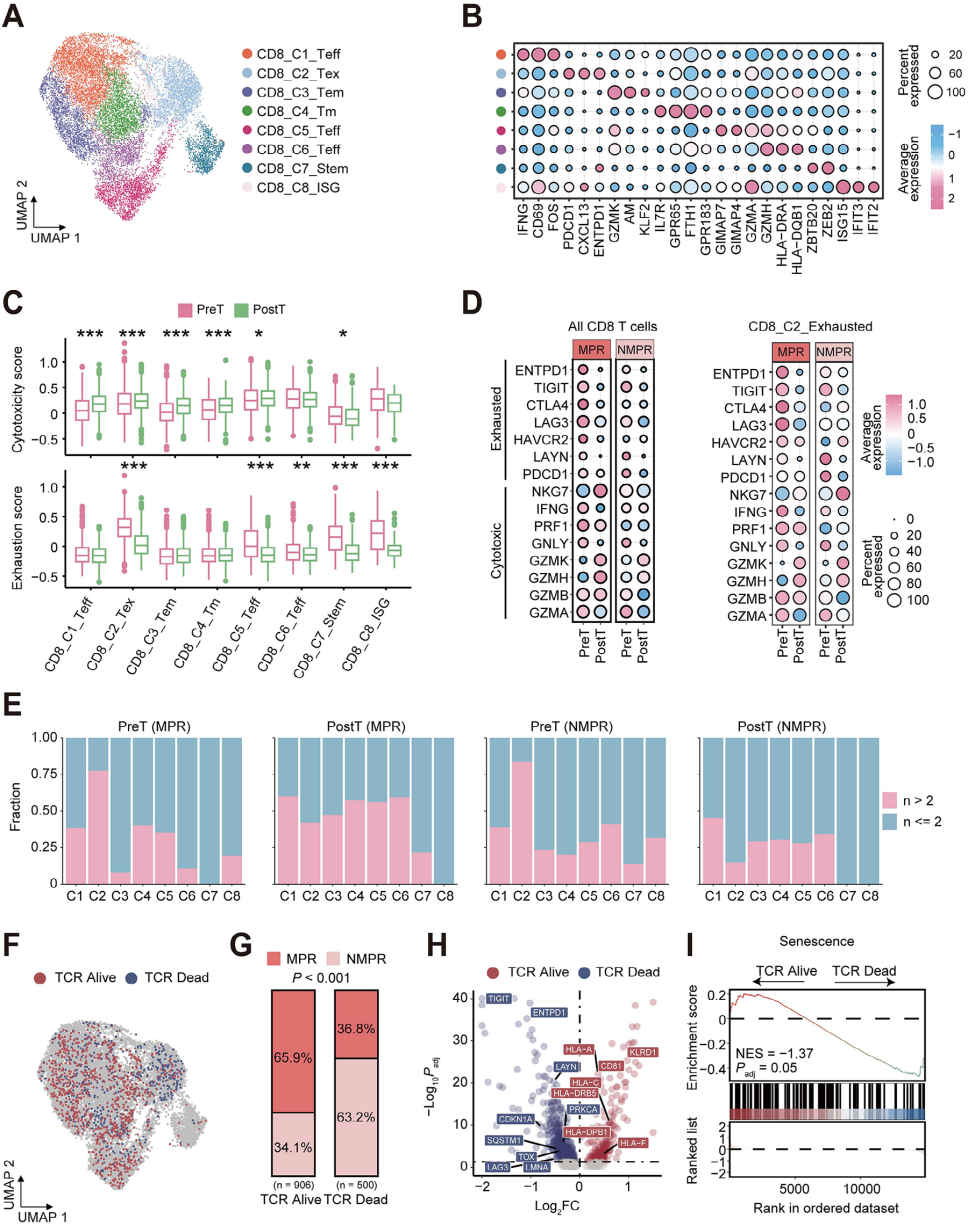
nICRT‐related changes in the CD8 T cell infiltration and cytotoxicity. (A) UMAP showing 8 CD8 cell subtypes. (B) Dotplot displaying the expression levels of the selected markers in different CD8 cell subtypes. Dot size reflects the fraction of expressing cells, and the colors denote normalized gene expression levels. (C) Boxplots of the cytotoxic and exhausted signature scores for different CD8 cell subtypes before and after treatment. *p*‐values were calculated by a two‐sided Wilcoxon rank‐sum test. (D) Dotplots showing the expression levels of exhausted and cytotoxic T cell marker genes across all CD8+ T (left panel) and CD8_C2_Exhausted (right panel) cells in tumor tissues from MPR and NMPR patients before and after treatment. (E) Fractions of TCR clones with different sizes (*n* > 2 and *n* ≤ 2) for different T cell clusters in different groups. (F) UMAP showing the two types of TCR characteristics in TCR shared CD8 cells. (G) Correlation between the two states of clonally shared TCR CD8 and treatment efficacy before and after treatment. *p‐*value was obtained using a two‐sided Chi‐squared test. (H) The volcano plot showing the differentially expressed genes between TCR_Alive CD8 and TCR_Dead CD8 cells. (I) GSEA showing senescence pathway was upregulated in TCR_Dead CD8 cells. The NES and FDR are included.

It is well known that CD8+ T cells play a pivotal role in immunotherapy. We performed unsupervised analysis using UMAP and identified 8 clusters of CD8+ T cells, including CD8_C1_Teff (effector T), CD8_C2_Tex (exhausted T), CD8_C3_Tem (effector memory T), CD8_C4_Tm (memory T), CD8_C5_Teff, CD8_C6_Teff, CD8_C7_Stem and CD8_C8_ISG (Figure 7A,B). Interestingly, CD8_C7_Stem cells had significant upregulation of a series of transcription factors, such as *STAT3*, *ZBTB20*, *FOXO1*, *BCL2*, *ZEB2*, and *TOX*, exhibiting stem‐like characteristics. CD8_C1_Teff displayed the highest activation and TCR signaling functional score, CD8_C2_Tex had the highest exhaustion gene signature, CD8_C5_Teff and CD8_C6_Teff displayed the highest cytotoxicity gene signature, while CD8_C7_Stem had the highest naive gene signature (Figure S10A).

During the progression from normal to tumor tissues, CD8+ T cells not only enhanced their cytotoxic function but also displayed elevated levels of exhaustion. After nICRT treatment, the abundance of exhausted CD8+ T cells significantly decreased (Figure 7C; Figure S10B), and the expression levels of a series of exhaustion molecules within this subset also decreased (Figure 7D). We also observed a significant increase in the proportions of several memory and effector CD8 clusters after nICRT treatment, particularly in MPR patients (Figure S10C). In addition, we found that the cytotoxicity gene signature score of CD8+ T cells significantly increased after treatment in the MPR patients, while no change was observed in the NMPR patients (Figure S10B). These findings demonstrate robust immune reactivation specifically in treatment‐responsive MPR patients.

### 2.7 Shared CD8+ T Cells in NMPR Patients Exhibit Heightened Senescent State

Further extraction of single‐cell TCR information from T lymphocytes showed no significant bias in terms of TCR quantity, specificity rearrangement events, or diversity across different populations (Figure S11A,B). T cells with TCR data across tissues and treatment time points were shown in the UMAP plots (Figure S11C). There is minimal overlap of TCR clones between tumor tissues before and after nICRT treatment, with most clones being newly generated (Figure S11D). Upon recognition of tumor antigens, tumor‐reactive CD8+ T cells undergo significant clonal expansion, forming a highly clonal population that enhances their anti‐tumor capacity [33, 34]. Therefore, we examined the clonal expansion patterns of different CD8+ T cell subtypes in ESCC during nICRT and found that a greater proportion of high‐expansion clones (*n* > 2 cells) were predominantly present in CD8+ Tex and Teff cells (Figure 7E). This result highlights the significant role of CD8+ Tex and Teff cells as CD8+ T cells specific to tumor antigens. Notably, MPR patients showed greater clonal expansion of CD8+ Teff and stem cells compared to NMPR patients following treatment (Figure S12A). The clonal expansion index of CD8+ Teff and stem cells in post‐treatment MPR patients was found to be higher than that in NMPR patients (Figure S12A), aligning with the observation that CD8+ Teff cells in MPR patients were highly activated. Furthermore, to trace the lineage transitions among CD8 cell phenotypes, we measured the level of clonotype overlap between each primary phenotype and all other phenotypes. We observed significant clonotype overlaps among CD8+ T cell clusters, with more active phenotype transitions in post‐treatment MPR patients, especially between CD8+ Tem or CD8+ Teff and CD8+ Stem cells (Figures S10D and S12B,C).

Next, we investigated the fate of T cells capable of withstanding treatment by tracking clonotypes that persisted from baseline. Although numerically rare, these clones provided a valuable window into the processes of long‐term immune memory and clonal exhaustion. We defined clones as TCR_Alive or TCR_Dead if the ratio of cells after treatment to the total (before + after) was greater than 0.3. Then, we identified 906 TCR_Alive cells and 500 TCR_Dead cells from a total of 7,451 CD8 cells that contain both TCR alpha and beta chains (Figure 7F). It was surprising that TCR_Alive cells and TCR_Dead cells showed distinct distributions. Specifically, TCR_Alive cells are predominantly enriched in MPR patients, while TCR_Dead cells are more concentrated in NMPR patients (Figure 7G), which might partially explain the difference in treatment efficacy between the two groups. Differential expression analysis revealed that TCR_Dead cells showed high expression of exhaustion markers and senescence‐related molecules (such as *CDKN1A*, *LMNA*, *ENTPD1*, and *TIGIT*), while TCR_Alive cells exhibited high expression of MHC‐related molecules (Figure 7H). We also revealed a significant expression elevation of genes in the senescence pathways in the TCR_Dead cells (Figure 7I). A sensitivity analysis confirmed the robustness of our findings, as the conclusions remained unchanged regardless of whether a post‐treatment frequency threshold of 0.4, 0.5, or 0.6 was applied (Figure S13). We further corroborated through mIHC that CD8 cells in NMPR patients had a more senescent phenotype, with notably elevated expression of CDKN1A (Figure S10E).

## 3 Discussion

The incorporation of immune checkpoint inhibitors (ICIs) into neoadjuvant therapy for locally advanced ESCC represents a significant advancement in addressing the ongoing challenges associated with high recurrence rates and inadequate pathologic responses observed with chemoradiotherapy (CRT) or chemotherapy (CT) alone. nICRT and nICT have shown promising short‐term efficacy in esophageal cancer. Preliminary data suggest that nICRT demonstrates higher pCR or MPR rates [19]. However, it remains uncertain whether elevated pCR rates will ultimately translate into improved OS rates. To our knowledge, this is the first clinical study to report 3‐year EFS and OS outcomes for nICRT in locally advanced ESCC. The observed 3‐year EFS rate of 68.2% and OS rate of 77.3% in our cohort compare favorably with historical controls: the NEOCRTEC5010 trial reported a 3‐year disease‐free survival rate of 68.9% with neoadjuvant chemoradiotherapy [7], while the JCOG1109 trial demonstrated a 61.8% 3‐year progression‐free survival (PFS) rate in its triplet chemotherapy arm. Notably, our 3‐year OS rates surpassed those reported in both the NEOCRTEC5010 (65.8%) and JCOG1109 (72.1%) [35]. Importantly, none of the patients achieving MPR in surgical specimens experienced recurrence or metastasis during the 3‐year follow‐up period. In contrast, those without MPR demonstrated a substantially higher 3‐year recurrence rate of 71.4%, indicating a poorer prognosis. These findings underscore MPR as a highly predictive early indicator for long‐term therapeutic outcomes.

Based on our findings, we conducted an efficacy stratification of patients who achieved MPR and NMPR, integrating scRNA‐seq, scTCR‐seq, and functional experiments to elucidate the mechanisms underlying treatment resistance in the nICRT regimen. A significant finding of our study is the notable increase in PRDM1+ tumor cells post‐treatment in patients exhibiting a poor response to nICRT. These cells exhibit marked lipid peroxidation, which is correlated with the upregulation of cysteine metabolism genes, such as MFSD12 and CTSB, by the transcription factor PRDM1. This leads to reduced intracellular cysteine levels, thereby impairing the clearance capacity of lipid peroxidation [36, 37]. Lipid peroxidation acts as a double‐edged sword in oncology [38, 39]. On one hand, it drives tumor progression through epigenetic modifications, disruption of membrane systems, and TME remodeling. Conversely, excessive accumulation of peroxidation products paradoxically triggers tumor cell death [40]. The transition between these pro‐tumorigenic and anti‐tumorigenic effects is conceptually governed by a “threshold”. However, defining a universal, quantitative value for this threshold is exceptionally challenging. It is highly context‐dependent, fluctuating based on cell type, the intensity and duration of the oxidative insult, and the cell's intrinsic antioxidant capacity. Nevertheless, to bridge these biological mechanisms to clinical application, we propose a ‘priming’ model to conceptualize this threshold in the context of therapeutic resistance. PRDM1+ malignant cells, driven by the transcriptional repression of cysteine metabolism genes (MFSD12 and CTSB), maintain a heightened basal level of lipid peroxidation. This metabolic state effectively ‘primes’ these cells by placing them significantly closer to the lethal lipid peroxidation threshold than their PRDM1‐negative counterparts or normal epithelial cells, which retain robust antioxidant reserves. Consequently, PRDM1+ cells possess a narrower therapeutic window. Therefore, a therapeutic insult that is a sub‐lethal to normal cells may be sufficient to push these ‘primed’ malignant cells beyond the tipping point for survival, triggering ferroptotic cell death. This model explains why targeting the ferroptosis pathway can selectively eliminate the resistant subpopulation despite the difficulty in defining a universal quantitative threshold. Our study identified that PRDM1‐positive cellular subsets exhibit pronounced lipid peroxidation signatures. These findings collectively suggest that PRDM1‐driven elevation of lipid peroxidation may serve as a critical determinant of poor prognosis in esophageal cancer patients undergoing nICRT. Ferroptosis, a recently identified non‐apoptotic cell death modality independent of caspase activation, is mechanistically driven by uncontrolled lipid peroxidation cascades that culminate in plasma membrane rupture and lytic cell death. Ferroptosis inducers, such as sorafenib and erastin, as well as GPX4 inhibitors like RSL3, are currently under clinical evaluation for solid tumors [41]. Targeting PRDM1‐high tumors with ferroptosis agonists may exploit their inherent peroxidation vulnerability—potentiating lethal lipid radical propagation while bypassing apoptosis resistance pathways. Such combination strategies warrant further investigation as a means to overcome treatment resistance in ESCC.

Additionally, we found that PRDM1 could bind to the CD47 promoter and upregulate CD47 expression, which interacts with its receptor SIRPA on LAMs, inhibiting their phagocytic function. The activation of the CD47–SIRPA axis prevents macrophages from effectively clearing tumor cells, contributing to nICRT resistance [42, 43]. Anti‐CD47 antibodies are being tested in lymphomas and solid tumors [44, 45], though ESCC‐specific data are limited. Our findings linking the CD47–SIRPA axis to therapy resistance support the potential of Anti‐CD47 antibodies in ESCC.

The IL1A–IL1R2 signaling pathway, modulated by PRDM1, has been identified as a pivotal mechanism for immunosuppression. The activation of IL1A by PRDM1 leads to the stimulation of IL1R2+CD4+ Tregs, which in turn inhibit antitumor immune responses by enlisting exhausted CD8+ T cells. IL‐1 α, recognized as a multifunctional cytokine derived from tumor cells, is pivotal in modulating the TME. Recent studies have demonstrated that IL‐1A contributes to the establishment of an immunosuppressive network within this microenvironment by directly influencing the activities of Tregs, M2 macrophages, and DCs, in addition to indirectly enhancing the expression of immune checkpoint molecules, including PD‐L1 [46, 47]. Targeting IL1A or IL1R2—through neutralizing antibodies or receptor blockers—could disrupt this pathway and enhance immune responsiveness. While IL1R2 antagonists have demonstrated potential in various cancer therapies, their efficacy in overcoming resistance during nICRT for ESCC necessitates additional investigation.

In patients with NMPR, the CD8+ T cells, which are crucial for tumor eradication, display signs of senescence, as indicated by increased levels of CDKN1A. Conversely, in MPR patients, there exists a subpopulation of CD8+ T cells that exhibit stem cell‐like properties. This subpopulation functions as a reservoir for cytotoxic CD8+ T cells, facilitating the continuous generation of these effector cells and thereby enhancing anti‐tumor immune responses. These cells, characterized by high expression of transcription factors like TCF1 and BCL6, retain stemness and proliferative capacity, providing a sustained source of CD8+ Teff cells [48, 49]. Our findings show that baseline abundance of stem‐like CD8+ T cells correlates with treatment benefit, emphasizing their importance in shaping antitumor immunity. However, the failure of these cells to overcome PRDM1‐driven immunosuppression highlights the need for combination strategies to bolster their activity.

While PRDM1 is well‐characterized in immune cells (T and B cells) [50, 51], our study uncovers a novel role for PRDM1 in ESCC epithelial cells, where its expression is linked to therapy resistance. Our data suggest this occurs through the modulation of both cysteine metabolism (via MFSD12 and CTSB) and immune checkpoints (CD47, IL1A). This apparent contradiction—its tumor‐suppressive role in prior studies versus its therapy‐resistant function here—likely stems from cell type‐specific actions. For instance, PRDM1 suppresses pro‐inflammatory cytokines in immune cells but activates immunosuppressive signals in epithelial cancer. Parallel tissue‐specific regulation is observed in hepatocellular carcinoma, where PRDM1 upregulates PD‐L1 via the USP22–SPI1 axis [51]. Our work expands PRDM1's biological repertoire, positioning it as a dual metabolic‐immune therapeutic target.

Based on these findings, we propose potential new strategies to reverse nICRT treatment resistance: (1) Ferroptosis Induction: Combining ferroptosis inducers (e.g., sorafenib, erastin) with nICRT could enhance ferroptosis in PRDM1+ tumor cells, exploiting their metabolic vulnerabilities. (2) Immune Checkpoint Blockade: Targeting the CD47‐SIRPA and IL1A‐IL1R2 axes with anti‐CD47 antibodies or IL1R2 blockers may reverse immunosuppression by restoring macrophage function and inhibiting Treg recruitment.

We acknowledge several limitations in the present study. First, the modest cohort size (*n* = 22 total; *n* = 9 scRNA‐seq) limits statistical power and necessitates validation in larger cohorts. Second, exclusive inclusion of Han Chinese patients (77.3% male) restricts generalizability across ethnic/gender groups due to potential biological and environmental heterogeneity. Third, our functional validation relied on in vitro co‐culture systems that, while informative, have inherent constraints. The use of the THP‐1 cell line does not fully capture the diverse functional states of primary tumor‐associated macrophages, and PBMC‐derived CD8+ T cells may not entirely replicate the phenotype of chronically stimulated tumor‐infiltrating lymphocytes. Therefore, these experiments should be interpreted as valuable proof‐of‐concept models that support the hypotheses generated from our patient data. Finally, although in vitro experiments provided mechanistic insights, they cannot fully replicate the complexity of the in vivo tumor ecosystem. The functional role of PRDM1‐mediated epithelial immune remodeling requires further verification in animal models and clinically diverse patient populations. In addition, the long‐term efficacy and safety of potential therapeutic strategies, such as ferroptosis inducers or CD47 inhibitors, should be carefully evaluated in future translational and clinical studies.

## 4 Conclusion

We have integrated scRNA‐seq, scTCR‐seq analysis, and functional assays and uncovered a malignant cell cluster with high PRDM1 protein expression as the crucial component contributing to nICRT resistance. In ESCC patients with poor response to nICRT treatment, PRDM1+ malignant cells interact with Treg cells and LAMs through the IL1A–IL1R2 and CD47–SIRPA pairs to remodel the epithelial immune environment for promoting immunosuppression. The key regulatory steps (e.g., ferroptosis sensitivity of tumor cells, cell‐cell interaction between tumor cells and Treg cells or LAMs) are all affected by PRDM1. In summary, we propose potential new strategies to reverse nICRT treatment resistance: (1) Combining ferroptosis inducers in combination with nICRT to enhance ferroptosis in PRDM1+ tumor cells. (2) Use inhibitors or antibodies of IL1A or CD47 to block the interaction between PRDM1+ tumor cells and Treg cells or LAMs, reversing the immunosuppressive state.

## 5 Experimental Section

### 5.1 Study Design, Participants, and Sample Collection

We initiated a non‐randomized, single‐arm, single‐center, phase‐II trial to investigate the efficacy, feasibility, and safety of the nICRT in the treatment of locally advanced ESCC. Patients underwent esophagectomy after neoadjuvant therapy completion between May 2019 and March 2022 at the Zhejiang Cancer Hospital, China. This study was conducted in accordance with the Declaration of Helsinki (as revised in 2013). All patients provided written informed consent for their enrollment in the study, and the study was approved by the Ethics Committee of the Zhejiang Cancer Hospital, China (Nos. IRB‐2019‐38). The study was registered with ClinicalTrials.gov, NCT03940001. Complete eligibility criteria and detailed information have been published [19].

Based on the above clinical trial, we obtained surgically removed 26 samples (normal tissues, adjacent non‐cancerous tissues, pre‐ and post‐treatment tumor tissues) from 9 patients at the Zhejiang Cancer Hospital, China (between May 2019 through March 2022, Table S2). All sample collection procedures followed the established protocols in clinical practice, and written informed consent had been secured. For the tumor samples intended for scRNA‐seq, the pre‐ and post‐treatment specimens were matched by site and collected from the same primary esophageal tumor lesion, with tumor samples obtained before treatment through endoscopic biopsy and after treatment via surgical resection. During the early phase of the trial, six patients were not biopsied because the study team prioritized safety monitoring before introducing additional sampling. In subsequent cases, tumor tissue collection was attempted whenever clinically feasible. Several patients were excluded due to endoscopic concerns regarding a high risk of bleeding, two samples yielded insufficient tissue for single‐cell sequencing, and two failed during single‐cell suspension preparation. Ultimately, nine representative cases were successfully processed for scRNA‐seq analysis, including five from the MPR group and four from the NMPR group.

### 5.2 Cell Culture

Human esophageal squamous cancer cell lines (KYSE‐30 and KYSE‐150) were maintained in DMEM (C11995500BT, Gibco, Grand Island, NY, USA) enriched with 10% fetal bovine serum (FBS‐CS500, NEWZERUM, New Zealand). Cultures were incubated at 37°C in a humidified atmosphere containing 5% CO_2_ to replicate physiological conditions. Penicillin‐streptomycin solution (100 IU/mL) (MP Biomedicals, Irvine, CA, USA) was included as required. Experiments were conducted when cells reached 70%–80% confluence, ensuring they were in an optimal growth phase.

### 5.3 Sample Preparation, Tissue Dissociation, and Cell Viability Examination

Tissue samples were diced into approximately 1‐mm^3^ segments. The tumor dissociation kit from Miltenyi Biotech was utilized according to the manufacturer's guidelines to dissociate all tissue samples. The resulting cell suspensions were sequentially filtered through 70‐ and 30‐µm cell strainers, followed by centrifugation at 300 × g for 10 min. After removing the supernatant, the cell pellet was resuspended in red blood cell lysis buffer from Miltenyi Biotech and incubated on ice for 2 min to eliminate any remaining red blood cells. Following two washes with 1 × phosphate‐buffered saline (PBS), the cell pellets were resuspended in RPMI‐1640 medium supplemented with 5% fetal bovine serum (FBS). Cell viability was assessed using the Countstar Rigel S2 cell counter from Countstar. The cell suspensions were mixed with AO/PI staining solution (Countstar; 12 µL:12 µL) at a 1:1 ratio, immediately placed into a disposable counting chamber, and analyzed with the Countstar Rigel S2. Parameters such as cell viability, concentration, and aggregation rate were evaluated and documented.

### 5.4 scRNA‐seq Library Preparation and Sequencing

scRNA‐seq was performed using the 10 × Genomics Chromium Platform, which included the isolation of single cells, amplification of complementary DNA (cDNA), and preparation of the library. First, the concentration of the single‐cell suspensions was assessed with the Countstar Rigel S2 (Countstar) and then loaded onto a Chromium Controller to generate single‐cell gel bead‐in‐emulsions (GEMs). Following this, the scRNA‐seq libraries were created using the Chromium Next GEM Single Cell 5ʹ Library & Gel Bead Kit (PN: 1000263, 10 × Genomics). The single‐cell GEMs enabled the production of barcoded cDNA through reverse‐transcription PCR, which was then purified with Dynabeads MyOne SILANE magnetic beads (PN: 2000048, 10 × Genomics). After this, cDNA amplification was carried out using the Amplification Master Mix kit (PN: 1000244, 10× Genomics), followed by purification and target enrichment with the Beckman Coulter SPRIselect reagent. The concentration and quality of the cDNA libraries were assessed using the Agilent Bioanalyzer High Sensitivity DNA kit. For library pooling, 50 ng of each sample library in 20 µL was mixed with 30 µL of Fragmentation Mix (PN: 2000091, 2000090, 10 × Genomics). The barcoded library was then sequenced on the NovaSeq6000 (Illumina) S2 flow cell (100 cycle kit) in a 26 × 91 run configuration with an 8‐bp index (read 1). To reduce batch effects, all libraries were generated using the same kit version and adhered to the identical protocol. Ultimately, the libraries were sequenced on the same NovaSeq6000 (Illumina) flow cell and analyzed together.

### 5.5 ScRNA‐seq Data Analysis

The Cell Ranger Single‐Cell toolkit was applied to align reads to the human reference genome GRCh38 (https://cf.10xgenomics.com/supp/cell‐exp/refdata‐gex‐GRCh38‐2020‐A.tar.gz) and generated the preliminary unique molecular identifiers (UMIs) matrix. The R package Seurat was used for scRNA‐seq downstream analyses. We performed quality control to filter out low‐quality cells based on two metrics: 1) the number of expressed genes larger than 300 and lower than 8,000; 2) the cells with less than 20% mitochondrial RNA content. The R package DoubletFinder [52] was applied to remove potential doublets in each sample. Finally, we identified 30 452 genes and detected 120 665 cells from 26 samples. We then normalized the “NormalizeData” function to perform the library‐size correction and logarithm transformation. We identified highly variable genes using the “FindVariableFeatures” function and scaled expression values regressing out the cell cycle gene score. We next performed principal component analysis (PCA) linear dimensionality reduction based on the scaled data and retained 30 principal components. The R package Harmony was used to adjust batch effects between different samples and integrate the gene expression matrices of all samples. The dimensionality of the dataset was further reduced using the Uniform Manifold Approximation and Projection (UMAP) with Seurat RunUMAP function. Next, the “FindNeighbors” function was used to construct a K‐nearest‐neighbor graph and the most representative principal components were used to determine different cell types with the “FindCluster” function. We annotated cell types and 9 clusters were identified based on expression of these maker genes: *CD3D* and *CD3E* for T cells, *COL1A1* and *COL1A2* for fibroblasts, *EPCAM* and *KRT5* for epithelial cells, *CD79A*, *MS4A1*, and *MZB1* for B cells, *CD86* and *LYZ* for myeloid cells, *CSPG4* and *ABCC9* for pericyte cells, *TPSAB1* and *TPSB2* for mast cells, *VWF* and *PECAM1* for endothelial cells, *CSF3R* for neutrophil cells.

### 5.6 scTCR‐seq Data Analysis

Cell Ranger Single‐Cell toolkit (version 7.0.1) was also applied to align TCR‐seq reads to the GRCh38 human reference genome (refdata‐cellranger‐vdj‐GRCh38‐alts‐ensembl‐7.1.0) and assemble TCR sequences. The TCR sequences containing full‐length, paired α and β TCR chains were preserved for further analysis. Each unique CDR3 (the complementarity‐determining region 3) sequence of TCR α or β chains was defined as a clonotype, and T cells that shared identical clonotypes were regarded as originating from the same cell clone. The filtered contig annotation csv file for TCR was used as an input for the scRepertoire package (1.8), to perform the clonotype analysis, CDR3 distribution, and repertoire overlap. The Startrac package was used to calculate the Expansion index between clusters.

### 5.7 Differential Expression

To annotate the cell type and state, differentially expressed genes (DEGs) were identified for each cell cluster using the “FindAllMarkers” function (multiple condition comparisons) with default parameters (min.pct = 0.25, logfc.threshold = 0.25). “FindMarkers” function was used to calculate differentially expressed genes under two condition comparisons, with the parameters (min.pct = 0.001, logfc.threshold = 0.001).

### 5.8 Definition of Gene Signature Score

CD4‐related and CD8‐related gene sets were obtained from Chu Y et al. [53]. Myeloid‐related gene sets were obtained from previous studies [54, 55, 56]. Cell type‐specific gene signatures of Epi_C4 (PRDM1), CD4_C2_Treg1, and Mac_C5 (TREM2) were defined as the top 40 DEGs ranked by transformed ratio of the normalized gene expression in each cluster. Lipid‐associated macrophages gene signatures were collected from Jaitin DA et al. [31] and Dib et al. [32]. A new senescence gene set was obtained from the previous study [57].

### 5.9 Calculation of Gene Signature Score

Curated gene sets related to T cell functional states were obtained from previous studies. The signature score of Epi_C4 (PRDM1), CD4_C2_Treg1, and Mac_C5 (TREM2) in scRNA‐seq datasets was calculated by Seurat's AddModuleScore function, while bulk RNA‐seq related signatures were performed using the gsva method in the GSVA R package.

### 5.10 Tissue Enrichment Analysis

For each cell subtype, we quantify the preference across different tissues by calculating the Ro/e as previously described [58]. Specifically, Ro/e was the ratio of the observed cell numbers over the expected cell numbers of a given combination of cell subtype and tissue, the expected cell numbers of each cell subtype in each tissue were obtained from the Chi‐square test, and Ro/e > 1 for a cell subtype in a tissue indicated preference of this cell subtype in this tissue.

### 5.11 Single‐Cell Copy Number Variation Analysis

Copy number variations of each cell were assessed with the R package inferCNV (provided at https://github.com/broadinstitute/inferCNV). Other parameters were set as default. All T cells, myeloid cells, and B cells were set as references.

### 5.12 Cell Developmental Trajectory Analysis

RNA velocity analysis was conducted using velocyto [59] and scVelo [60]. We used the 10× velocyto pipeline to count spliced and non‐spliced reads for each sample from cellranger‐generated BAM files. The single‐cell trajectory analysis of cancer cell subtypes was performed with Monocle 2 [61] using DDR‐Tree and default parameters.

### 5.13 Gene Set Enrichment Analysis

For scRNA‐seq datasets, we performed the gene set enrichment analysis for select cell subtypes by the GSEA function from R package clusterProfiler [62], with Reactome GO, KEGG, and Hallmark pathways from MSigDB [63], and some collecting signatures. For the published bulk RNA‐seq datasets, the DEGs were identified by DEseq2 [64] and used for GO and KEGG enrichment analysis by the enrichGO and enrichKEGG function.

### 5.14 Cell‐Cell Interaction Analysis

We used CellChat2 [65] with default parameters to calculate cell–cell interaction patterns among cell populations in the microenvironment. The netVisual_circle function was applied to visualize the communication strength between different cell types, and the netVisual_bubble function was applied to visualize communication probabilities by ligand‐receptor pairs.

### 5.15 Reagents and Antibodies

Ferrostatin‐1 (S7243) was purchased from GLPBIO (Montclair, CA, USA). Deferoxamine mesylate (S5742), N‐acetylcysteine (S1623), RSL3 (S8155), and Erastin (S7242) were purchased from Selleckchem (Houston, TX, USA). The following antibodies were used in our study. The anti‐PRDM1/BLIMP1 (61168) antibody was purchased from Active Motif (Carlsbad, CA, USA). The anti‐MFSD12/C19orf28 (PA5‐59891) antibody was purchased from Thermo Fisher (Waltham, MA, USA). Antibodies against Cathepsin B (12216‐1‐AP), CD47 (20305‐1‐AP), and IL‐1α (16765‐1‐AP), as well as horseradish peroxidase (HRP)‐conjugated goat anti‐mouse IgG (SA00001‐1) and HRP‐conjugated goat anti‐rabbit IgG (SA00001‐2), were purchased from Proteintech (Rosemont, IL, USA).

### 5.16 Plasmids

The plasmid pCMV‐PRDM1(human)‐3×FLAG‐SV40‐Neo (P37371) used for transient transfection to overexpress the PRDM1 transcription factor, along with the empty vector control plasmid pSin‐EF2‐bleo (P60343), was purchased from MiaoLingBio (Wuhan, China). Transfection of all plasmids into the cell lines was performed with Lipofectamine 3000 (Invitrogen, LC3000015) and Opti‐MEM (Gibco, 31,985,070), in accordance with the manufacturer's guidelines.

### 5.17 Cell Viability Assays

Cells were initially plated in a 96‐well plate, with three replicate wells allocated per group, and then incubated overnight at 37°C within a cell culture incubator. Following this, the cells were subjected to treatment with the specified drugs for a duration of 72 h. Subsequently, MTT was introduced into each well, and the plates were incubated at 37°C for an additional 4 h. After incubation, the supernatant was aspirated, and the resulting formazan crystals were dissolved in 200 µL of DMSO. Ultimately, cell viability was assessed by measuring the absorbance at 490 nm using a microplate reader (Epoch Biotek, USA).

### 5.18 Determination of Lipid Peroxidation

Cells were seeded in six‐well plates at a density of 3 × 10^5^ cells per well. On the second day, the cells were treated with the indicated compounds and then collected, stained with 5 µm BODIPY 581/591 C11 (Invitrogen, D3861) at 37 °C for 30 min and analyzed by flow cytometry. For BODIPY 581/591 C11 staining, only the FITC channel was monitored. The fluorescence intensity of the FITC channel was analyzed for each sample. Cells undergoing lipid peroxidation were defined as cells with a high FITC fluorescence intensity. The gate to define increased FITC fluorescence intensity was established based on the fluorescence intensity distribution of cancer cells treated with DMSO at the same concentration as the vehicle control group receiving maximal drug dosage, which were assumed to have minimal lipid peroxidation. Lipid reactive oxygen species‐positive cells were identified as those exhibiting FITC fluorescence intensity exceeding approximately 85% of the vehicle‐treated control population. At least 5,000 cells were analyzed in each group and all experiments were repeated at least three times. Data analysis was performed using FlowJo V10.9.0.

### 5.19 Cysteine Concentration Assay

The Protein Cysteine Assay Kit with DTNB was purchased from Beyotime (S0145S). According to the kit instructions, cells were lysed with cell lysis buffer and centrifuged at 12 000 × g for 10 min at 4°C to collect supernatants. Protein concentration was determined using Pierce BCA Protein Assay Kit (ThermoFisher, 23227). GSH standards (0–4 mm) and samples were prepared, and Ellman's Reagent Solution was freshly made. Samples were treated with 100 µL Denaturing Buffer and 2 µL Reduction Buffer, then incubated at 25°C for 1 h to reduce disulfide bonds. Following reduction, 1 mL Protein Precipitation Solution was added to precipitate proteins, and the mixture was incubated at 25°C for 1 h. The samples were then centrifuged at 5000 × g for 15 min at 4°C to pellet the proteins. The supernatant was discarded, and the pellet was washed twice with 500 µL Protein Precipitation Solution, centrifuging at 5000 × g for 15 min at 4°C each time. Finally, the pellet was resuspended in 30 µL Assay Buffer. Reactions were set up in a 96‐well plate with Ellman's Reagent Solution, incubated at room temperature for 15 min, and absorbance was measured at 412 nm using an Epoch microplate reader (Biotek, USA). Thiol concentration was determined from the standard curve, and cysteine concentration was calculated based on protein concentration.

### 5.20 GSH/GSSG Assay

The GSH and GSSG levels were determined using a GSH and GSSG Assay Kit (Beyotime, S0053) according to the manufacturer's instructions. Briefly, for total glutathione analysis, cell samples were lysed by freeze‐thaw cycles in liquid nitrogen and a 37°C water bath after being suspended in Protein Removal Reagent M solution. Erythrocyte or plasma samples were prepared by centrifugation of whole blood and subsequent washing with PBS. The lysates were centrifuged at 10 000 × g for 10 min at 4°C, and the supernatants were collected for analysis. For GSSG measurement, GSH was removed from the samples using diluted GSH Removal Buffer and GSH Removal Reagent working solution, followed by incubation at 25°C for 60 min. The total glutathione and GSSG contents were quantified by preparing standard curves with GSSG standards and measuring the absorbance at 412 nm using a microplate reader (Epoch Biotek, USA). The GSH content was calculated by subtracting the GSSG content (multiplied by 2) from the total glutathione content.

### 5.21 siRNA Transfection

The siRNA sequences we used are listed in Table S3. GenePharma synthesized all siRNAs, which were then introduced into the cells utilizing siRNA‐mate (G04003‐1) purchased from GenePharma and Opti‐MEM in accordance with the manufacturer's guidelines.

### 5.22 Quantitative Reverse Transcription–PCR (qRT–PCR)

Total RNA was isolated from cultured cells using the Cell RNA Quick‐Extraction Kit (GOONIEBIO, 400‐100). Subsequently, reverse transcription was carried out with the HiScript II Q RT SuperMix for qPCR with gDNA wiper (Vazyme, R223‐01). Real‐time PCR was conducted using ChamQ SYBR qPCR Master Mix (Vazyme, Q311‐03) on a Light Cycler 480 instrument (Roche Diagnostics) in accordance with the manufacturer's protocols. Each gene was analyzed in triplicate. The primers used in the amplification process targeting PRDM1/MFSD12/CTSB/CD47/IL1A were listed in Table S3. All primers used in this study were synthesized by Beijing Ribobio Biotechnology Co., Ltd. (Beijing, China).

### 5.23 Multiplex Immunohistochemistry Analysis

Multiplex immunohistochemistry was conducted using the PANO 7‐plex IHC kit (Panovue, Beijing, China). In detail, slides were incubated at 65°C for 2 h, deparaffinized with xylene, and rehydrated through a graded ethanol series (100%, 95%, 70%, 50%), followed by fixation in 10% neutral buffered formalin for 30 min. Antigen retrieval was performed using EDTA buffer (pH = 9.0, ZSGB‐Bio, Beijing, China) in a microwave, after which the sections were blocked to reduce non‐specific binding. Primary antibodie were sequentially applied, followed by incubation with horseradish peroxidase (HRP)‐conjugated secondary antibodies. Tyramide signal amplification (TSA) was performed after each primary antibody application to enhance signal detection. Subsequently, the sections were incubated with biotinylated rabbit polyclonal anti‐rabbit and rabbit anti‐mouse secondary antibodies, followed by treatment with HRP‐conjugated streptavidin according to the manufacturer's instructions (Panovue, Beijing, China). Biotinylated secondary antibodies were used for streptavidin‐linked alkaline phosphatase‐dependent chromogenic reactions and streptavidin‐linked fluorophores for immunofluorescence (excitation wavelengths: 480, 520, 570, 690, or 780 nm). Multispectral images were acquired using the Vectra Polaris Automated Quantitative Pathology Imaging System (Akoya Biosciences, Delaware, USA).

### 5.24 Indirect Coculture Systems

Two types of indirect co‐culture systems were established using 24‐well plates with transwell inserts (pore size: 0.4 µm; Corning Incorporated, Corning, NY, USA). For all setups, cancer cells were seeded in the lower chamber, while immune cells were placed in the upper chamber (the insert). In the first system, designed to assess the influence of Tregs, 1 × 10^5^ KYSE‐150 or KYSE‐30 cells were seeded into the lower chamber. Concurrently, a mixture of approximately 1 × 10^6^ CD8+ T cells and 1 × 10^6^ Tregs was seeded into the upper chamber. The co‐culture was maintained for 48 h. In the second system, designed to model the effects of LAMs, 1 × 10^5^ KYSE‐150 or KYSE‐30 cells were seeded into the lower chamber. Concurrently, a mixture of approximately 1 × 10^5^ LAMs and 1 × 10^6^ CD8+ T cells was seeded into the upper chamber. This co‐culture was also maintained for 48 h.

### 5.25 Flow Cytometry Analysis

Suspension cells in the upper chamber of the Transwell were stimulated with Leukocyte Activation Cocktail (BD Biosciences, San Jose, CA, USA) for 6 h. After stimulation, the cells were harvested using staining buffers (BD Biosciences, San Jose, CA, USA). To minimize background staining, the cells were pre‐incubated with 2 µg/mL Fc receptor blocker (Absin, China) for 15 min prior to antibody addition. For cell‐surface staining, the cells were labeled with anti‐CD8‐FITC (BioLegend, San Diego, CA) and anti‐PD1‐BV785 (BioLegend, San Diego, CA). The cells were incubated at 4°C for 30 min, followed by washing with fluorescence‐activated cell sorting (FACS) buffer. To enable intracellular staining, the cells were fixed and permeabilized using a fixation/permeabilization solution kit (BD Biosciences, San Jose, CA, USA). After fixation and permeabilization, the cells were stained with anti‐IFNG‐BV421 (BioLegend, San Diego, CA) and anti‐GZMA‐APC (BioLegend, San Diego, CA). The cells were incubated at 4°C for 30 min, then washed and resuspended in FACS buffer. The acquired data were analyzed using FlowJo 10.7 software.

### 5.26 The Acquisition of Tregs and CD8+ T cells

Naive CD4+ T cells were obtained from PBMCs with the EasySep Human Naïve CD4+ T Cell Isolation Kit (StemCell Technologies, Vancouver, CA, USA). Naive CD4 cells were cultured for 7 days using ImmunoCult Human Treg Differentiation Supplement (StemCell Technologies, Vancouver, CA, USA) to generate Tregs. Nuclear transcription factor FOXP3 is the definitive molecular marker for Tregs, but intracellular FOXP3 staining requires cell fixation and permeabilization, precluding subsequent functional assays that demand viable cell isolation. Importantly, multiple studies have demonstrated that the concurrent high expression of CD25 and low expression of CD127 closely correlates with FOXP3 expression and identifies T cell populations with bona fide suppressive activity, making this surface marker combination ideal for our functional experiments. We ultimately used the CD4+CD25+CD127 low flow cytometry gating strategy, ensuring both specific Treg enrichment and valid subsequent secretory factor analysis. Tregs were then confirmed by flow cytometry using anti‐CD4‐FITC and anti‐CD25‐PE antibodies. CD8+ T cells were obtained from EasySep Human CD8 Positive Selection Kit II (StemCell Technologies, Vancouver, CA, USA). CD8+ T cells were cultured for 7 days using ImmunoCult‐XF T Cell Expansion Medium (StemCell Technologies, Vancouver, CA, USA).

### 5.27 The Acquisition of LAMs

THP‐1 cells were seeded into 24‐well plates at a density of 2 × 10^5^ cells/mL and treated with PMA at a final concentration of 100 ng/mL. After 48 h of incubation, the THP‐1 cells adhered to the surface and displayed a macrophage‐like morphology. Subsequently, oxLDL (Yiyuan biotechnology, Beijing, China) was added to the culture at a final concentration of 50 µg/mL, and the cells were further incubated for 48 h to induce the formation of LAMs. The LAMs were identified by staining with Nile Red reagent and visualizing under a confocal microscope.

### 5.28 CUT&Tag‐seq and Data Analysis

KYSE‐30/KYSE‐150 cells (1 × 10^5^) were incubated with 10 µL Concanavalin A‐coated magnetic beads (Bangs Laboratories) for 10 min at RT. Bead‐bound cells were resuspended in primary antibody (Cat. No.: 9115S) buffer with 1:50 diluted primary antibody and incubated overnight at 4°C. After washing, cells were incubated with 1:100 diluted secondary antibody (Cat. No.: N269) for 60 min at RT, followed by 1:100 pA‐Tn5 adapter complex in dig‐300 buffer for 1 h. Tagmentation was performed in MgCl_2_‐containing buffer at 37°C for 1 h. DNA was purified using Tagment DNA Extract Beads. Libraries were amplified with NEBNext HiFi PCR Master Mix (14 cycles) and purified with XP beads. Sequencing was performed on an Illumina Novaseq6000 (150‐bp paired‐end). Paired‐end reads were aligned to the GRCh38 human reference genome (hg38) using Bowtie2 [66] version 2.5.4. Peak calling was performed using MACS2 (version 2.2.9.1) [67] with a threshold at *p*‐value of 0.001 as a cutoff based on input.

### 5.29 Statistical Analysis

The pre‐processing procedures for scRNA‐seq, scTCR‐seq, and CUT&Tag‐seq data are detailed in the respective methods sections. The detailed statistical test methods, data presentation, sample sizes, and *p‐values* used in this study were indicated in the corresponding legends and captions. Comparisons between two groups were performed using the Wilcoxon rank‐sum test. Box plots show the median (center line), 25th, and 75th percentiles. A *p*‐value < 0.05 was considered significant. Spearman's rank correlation analysis was used to assess associations between variables, with a weak‐to‐moderate association defined as R > 0.25 and statistical significance set at *p* < 0.05. Signature scores of cell clusters and collecting some genesets were calculated by GSVA and dichotomized at the median value or the best cut‐off point determined by surv_cutpoint function of the survminer R package for survival analyses. Survival curves were measured using the Kaplan–Meier method, and *p*‐values were calculated using a log‐rank test unless otherwise specified. EFS was defined as the time from treatment initiation to the first occurrence of progressive disease (RECIST v1.1), local or regional recurrence, distant metastasis, or death from any cause. Patients without an event were censored at the date of last follow‐up. OS was time to death from any cause, with survivors censored at the data cutoff (March 27, 2025). All survival analyses followed the intention‐to‐treat principle and were performed in the FAS, which included all 22 enrolled patients. The corresponding 95% CIs were calculated using the Brookmeyer–Crowley method (for median survival) and the exponential Greenwood formula (for survival rate). Given the relatively small sample size and low number of observed events, survival estimates should be interpreted as exploratory and hypothesis‐generating. Follow‐up time was calculated from initial treatment to last known alive date and is presented as median and IQR using descriptive statistics. All statistical analyses and visualizations were performed in R (V4.2.1) or Prism 8.0 (GraphPad).

## Author Contributions

D.S., Q.C., Y.J., and M.C. conceived and designed the entire project. M.C., D.S., Q.Z., and X. Li. designed and supervised the research. D.S., Y.J., Z.S., and R.C. prepared all samples for high‐throughput sequencing. Y.H. performed CUT&Tag‐seq. Y.H. and X. Li performed qRT‐PCR, cell migration assays, invasion assays, lipid peroxidation determination, GSH/GSSG ratio detection, cell viability assays, cell proliferation assays, and immunofluorescence staining. R.L. and Y.S. performed statistical and bioinformatics analyses of high‐through sequencing data. R.L. and X.L. were engaged in the analysis of public data. Q.Z. supervised all bioinformatics analyses. D.S., Y.J., Z.S., and M.C. were responsible for tissue sample preparation. D.S., R.L., Y.S., X. Liu, Y.H., K.C., X. Li, and M.C. prepared the manuscript, and all authors commented on the manuscript.

## Conflicts of Interest

The authors declare no conflicts of interest.

## Supporting information




**Supporting File 1**: advs73884‐sup‐0001‐SuppMat.pdf.

**Supporting File 2**: advs73884‐sup‐0002‐Tables.docx.

## Data Availability

The scRNA‐seq and scTCR‐seq data generated in this study have been deposited to Genome Sequence Archive (GSA) for Human (https://ngdc.cncb.ac.cn/gsahuman/) with an accession number of HRA010350. PRDM1 CUT&Tag‐seq datasets (accession number: GSE289774) can be obtained from the Gene Expression Omnibus database (GEO, https://www.ncbi.nlm.nih.gov/geo/). Bulk RNA‐seq datasets of the TCGA ESCC cohort can be downloaded from the XENA data portal (http://xena.ucsc.edu/) [68]. Public ESCC bulk RNA‐seq datasets (accession number: GSE23400 [69], GSE44021 [70], GSE38129 [71]) can be obtained from the Gene Expression Omnibus database (GEO, https://www.ncbi.nlm.nih.gov/geo/). The ESCC cohort with anti‐PD1 immunotherapy datasets was obtained from ESCC‐ORIENT‐2 [72]. The analysis code has been deposited on GitHub (https://github.com/sheyong111/escc_nICRT_manucript).
