# Supplementary material for: PRDM1+ Malignant Cells Mediate an Immunosuppressive Landscape and Resistance to Neoadjuvant Chemoradiotherapy and Immunotherapy in Esophageal Squamous Cell Carcinoma
Source: Adv Sci (Weinh). 2026 Jan 20;13(17):e15207. doi: 10.1002/advs.202515207 (PMC13042517; doi:10.1002/advs.202515207)
Supplement: Supplementary file 1 — Supporting File 1: advs73884‐sup‐0001‐SuppMat.pdf. [file ADVS-13-e15207-s002.pdf]

Figure S1

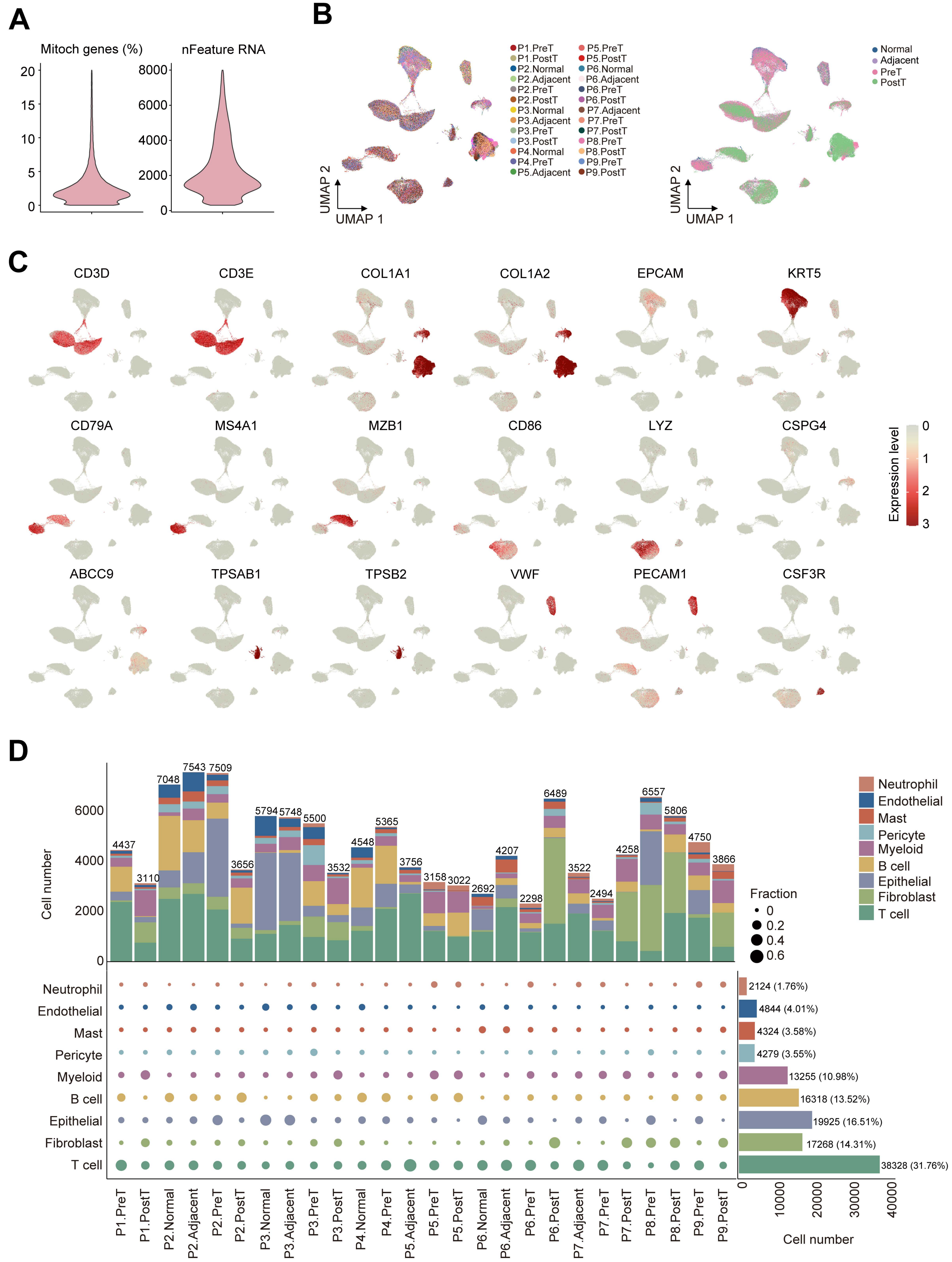

A
Mitoch genes (%)
nFeature RNA
B
UMAP 1
UMAP 2
P1.PreT
P1.PostT
P2.Normal
P2.Adjacent
P2.PreT
P2.PostT
P3.Normal
P3.Adjacent
P3.PreT
P3.PostT
P4.Normal
P4.PreT
P5.Adjacent
P5.PreT
P5.PostT
P6.Normal
P6.Adjacent
P6.PreT
P6.PostT
P7.Adjacent
P7.PreT
P7.PostT
P8.PreT
P8.PostT
P9.PreT
P9.PostT
Normal
Adjacent
PreT
PostT
C
CD3D
CD3E
COL1A1
COL1A2
EPCAM
KRT5
CD79A
MS4A1
MZB1
CD86
LYZ
CSPG4
ABCC9
TPSAB1
TPSB2
VWF
PECAM1
CSF3R
Expression level
D
Cell number
Fraction
Neutrophil
Endothelial
Mast
Pericyte
Myeloid
B cell
Epithelial
Fibroblast
T cell
4437
3110
7048
7543
7509
3656
5794
5748
5500
3532
4548
5365
3756
3158
3022
2692
4207
2298
6489
3522
2494
4258
6557
5806
4750
3866
2124 (1.76%)
4844 (4.01%)
4324 (3.58%)
4279 (3.55%)
13255 (10.98%)
16318 (13.52%)
19925 (16.51%)
17268 (14.31%)
38328 (31.76%)

1. Supplementary Figures

Figure S1, Supporting Information

Figure S1. scRNA-seq profiling of all cells in locally advanced ESCC. A) Histogram showing the total number of mitochondrial genes (left panel) and detected genes (right panel). B) UMAP plot showing different sample (left panel) and tissue origin (right panel) by color. C) UMAP plot visualizing the expression levels of marker genes, defined for 9 major cell clusters. D) Dotplot and barplot showing the proportions and cell numbers of each cell type across all sample.

Figure S2

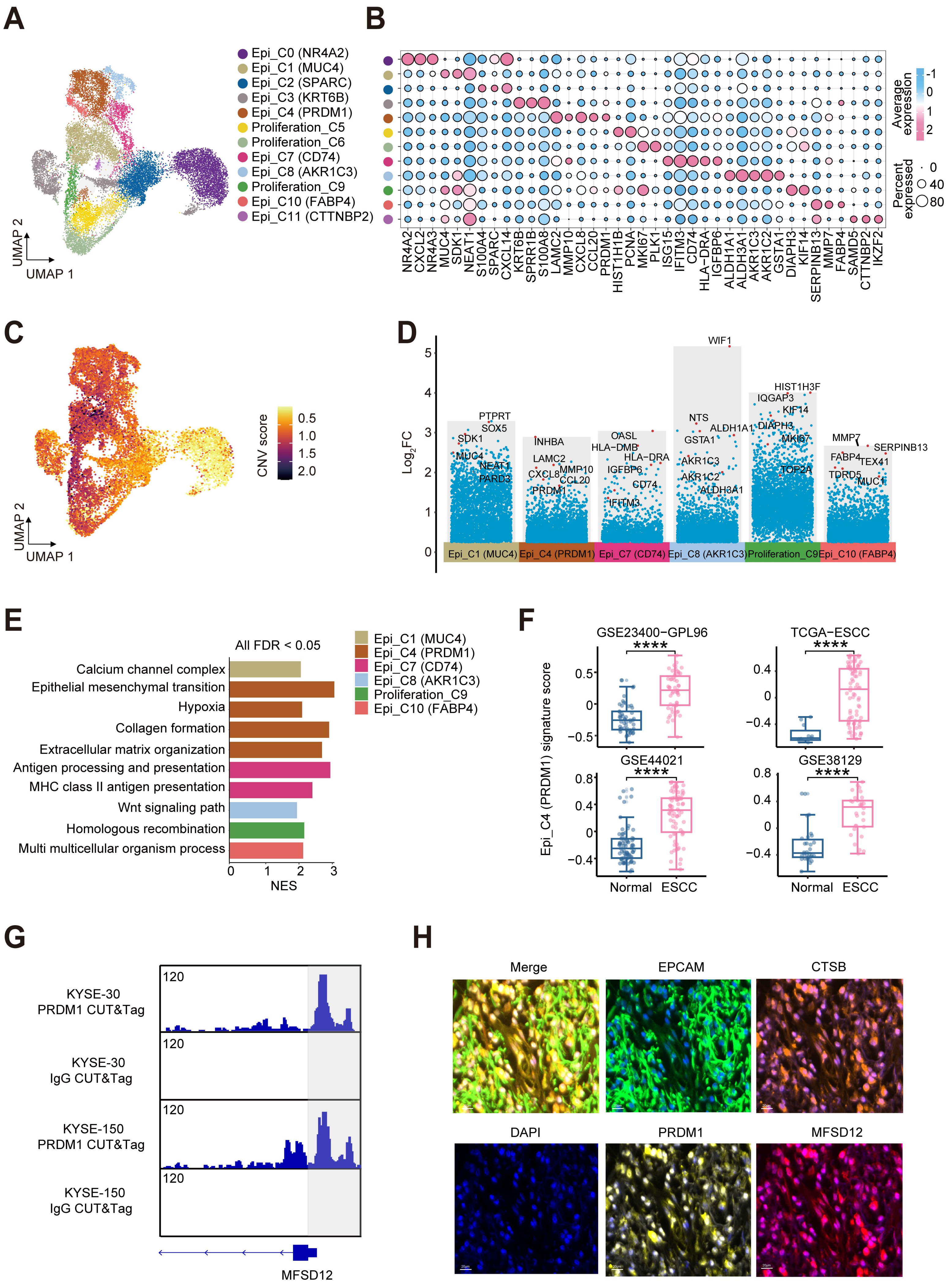
A
Epi_C0 (NR4A2)
Epi_C1 (MUC4)
Epi_C2 (SPARC)
Epi_C3 (KRT6B)
Epi_C4 (PRDM1)
Proliferation_C5
Proliferation_C6
Epi_C7 (CD74)
Epi_C8 (AKR1C3)
Proliferation_C9
Epi_C10 (FABP4)
Epi_C11 (CTTNBP2)
UMAP 1
UMAP 2
B
Average expression
Percent expressed
C
CNV score
D
Log2FC
Epi_C1 (MUC4)
Epi_C4 (PRDM1)
Epi_C7 (CD74)
Epi_C8 (AKR1C3)
Proliferation_C9
Epi_C10 (FABP4)
E
All FDR < 0.05
Calcium channel complex
Epithelial mesenchymal transition
Hypoxia
Collagen formation
Extracellular matrix organization
Antigen processing and presentation
MHC class II antigen presentation
Wnt signaling path
Homologous recombination
Multi multicellular organism process
NES
F
GSE23400−GPL96
TCGA−ESCC
GSE44021
GSE38129
Epi_C4 (PRDM1) signature score
Normal
ESCC
G
KYSE-30 PRDM1 CUT&Tag
KYSE-30 IgG CUT&Tag
KYSE-150 PRDM1 CUT&Tag
KYSE-150 IgG CUT&Tag
MFSD12
H
Merge
EPCAM
CTSB
DAPI
PRDM1
MFSD12

Figure S2, Supporting Information

Figure S2. A PRDM1+ malignant cell subcluster indeed existed in NMPR ESCC patients. A) UMAP showing 12 epithelial cell subtypes. B) Dot plot displaying the expression levels of the selected markers in different epithelial cell subtypes. Dot size reflects the fraction of expressing cells and the color denotes normalized gene expression levels. C) UMAP displaying the copy number variation (CNV) score of all epithelial cells. D) Representative upregulated genes of malignant cell subtypes based on DEG analysis. E) Enrichment of pathways in malignant cell subtypes. The x-axis represents the NES value of each pathway (FDR < 0.05 and NES > 1.5). F) Comparison of Epi_C4 signature scores between normal and primary ESCC samples (four published datasets), including the TCGA-ESCC cohort (N = 87), the GSE23400 cohort (N = 106), the GSE44021 cohort (N = 146), and the GSE38129 cohort (N = 60). ****$P < 0.0001$ as determined by two-tailed Wilcoxon rank-sum tests. G) Genome browser tracks of PRDM1 CUT&Tag-seq at the promoter region of MFSD12 in KYSE-30 and KYSE-150 cells. H) mIHC staining of EPCAM, PRDM1, CTSB, MFSD12, and DAPI in post-treatment tumor tissue sample of a representative NMPR patient. Scale bars: 20 µm.

Figure S3

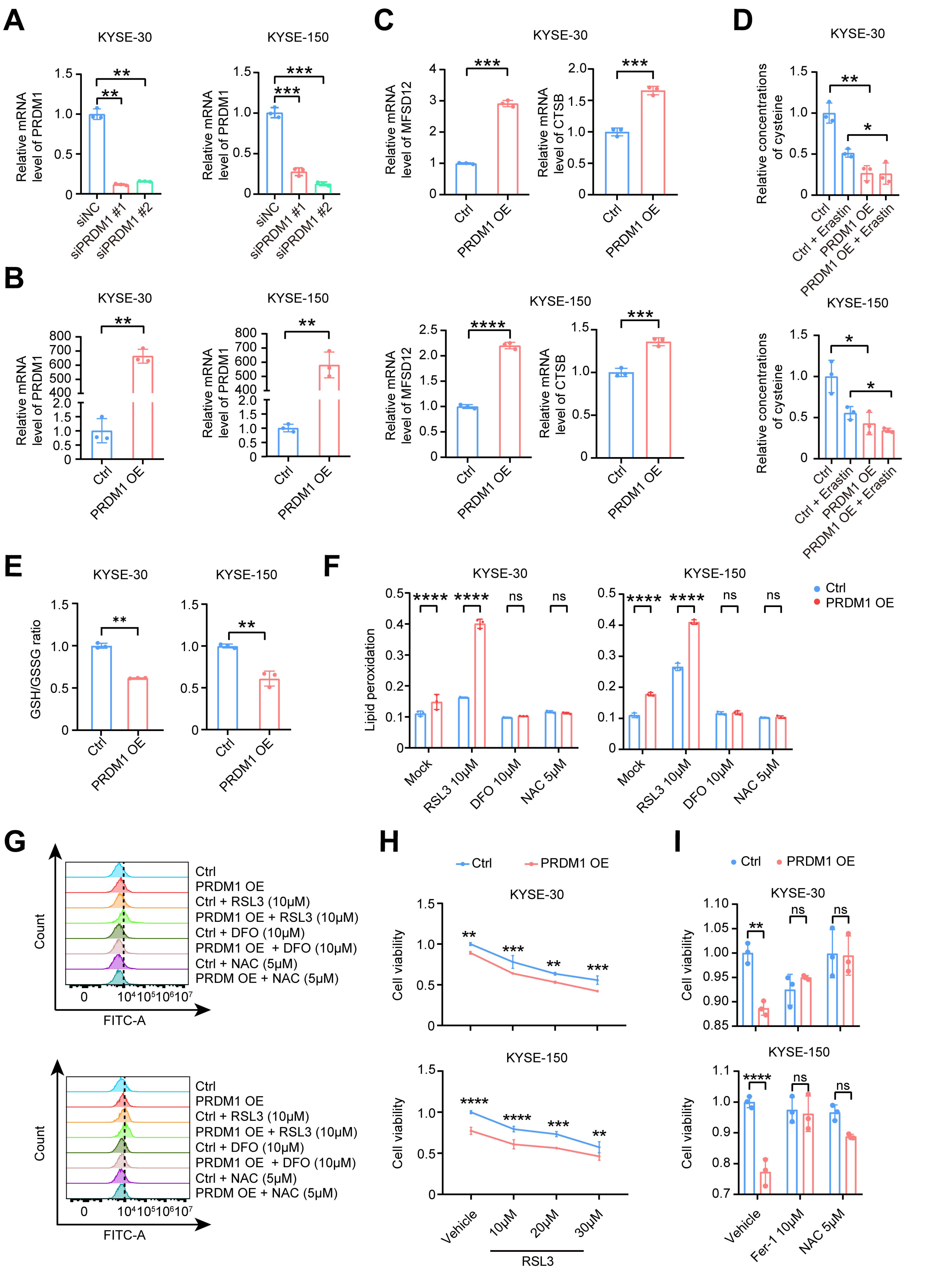

A
KYSE-30
KYSE-150
Relative mRNA level of PRDM1
siNC
siPRDM1 #1
siPRDM1 #2
B
Ctrl
PRDM1 OE
C
Relative mRNA level of MFSD12
Relative mRNA level of CTSB
D
Relative concentrations of cysteine
Ctrl + Erastin
PRDM1 OE + Erastin
E
GSH/GSSG ratio
F
Lipid peroxidation
Mock
RSL3 10μM
DFO 10μM
NAC 5μM
ns
G
Count
FIT C-A
Ctrl + RSL3 (10μM)
PRDM1 OE + RSL3 (10μM)
Ctrl + DFO (10μM)
PRDM1 OE + DFO (10μM)
Ctrl + NAC (5μM)
PRDM OE + NAC (5μM)
H
Cell viability
Vehicle
10μM
20μM
30μM
RSL3
I
Fer-1 10μM

Figure S3, Supporting Information

Figure S3. PRDM1 modulates cysteine metabolism and lipid peroxidation dynamics in ESCC cells. A) Relative *PRDM1* mRNA levels in KYSE-30 and KYSE-150 cells transfected with PRDM1-targeting siRNA (siPRDM1) or non-targeting control siRNA (siNC), quantified by qRT-PCR. B) *PRDM1* mRNA levels in cells transfected with PRDM1 overexpression plasmid (PRDM1 OE) versus empty vector control. C) Relative mRNA expression of *MFSD12* and *CTSB* in PRDM1-overexpressing cells. D) Relative cysteine concentration in cellular proteins was measured by colorimetric assay in untreated cells or cells exposed to 20 µM Erastin for 24 h. E) GSH/GSSG ratio in PRDM1 OE and control cells. F) Cell viability was assessed by MTT assay following treatment with DMSO (1:250) or four escalating concentrations of RSL3. G) Viability of cells treated with DMSO (1:1000), 10 µM Ferrostatin-1 (Fer-1), or 5 µM N-acetylcysteine (NAC). H) Percentage of cells with elevated lipid peroxidation after 24 h treatment with 10 µM RSL3, 10 µM deferoxamine (DFO), or 5 µM NAC versus DMSO control (1:1000), determined by BODIPY 581/591 C11 staining. I) Flow cytometry histograms showing lipid peroxidation levels (FITC-A intensity) in PRDM1-overexpressing cells under indicated treatments. Data are presented as mean ± standard error of the mean (SEM). *P* values were calculated by unpaired two-tailed Student's t-test (B, C, E, F, H and I) or one-way ANOVA (A and D), * $P < 0.05$, ** $P < 0.01$, *** $P < 0.001$, **** $P < 0.0001$; ns, not significant.

Figure S4

A

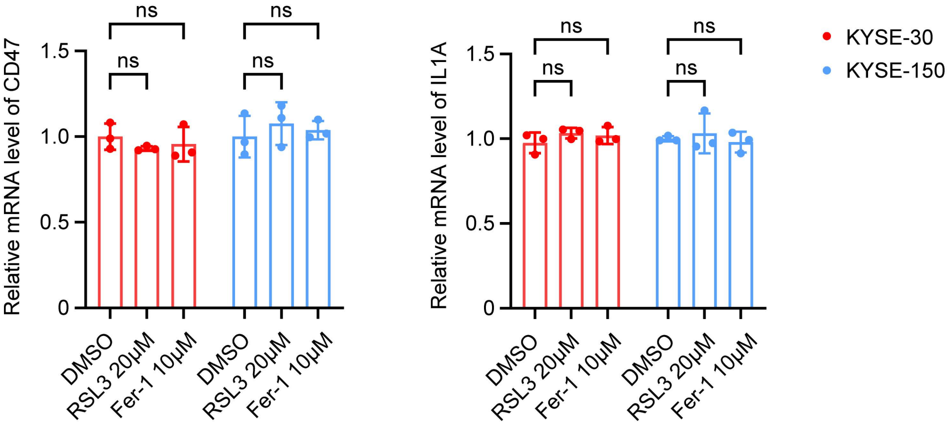

ns
ns
ns
ns
1.5
1.0
0.5
0
Relative mRNA level of CD47
DMSO
RSL3 20μM
Fer-1 10μM
DMSO
RSL3 20μM
Fer-1 10μM
ns
ns
ns
ns
1.5
1.0
0.5
0
Relative mRNA level of IL1A
DMSO
RSL3 20μM
Fer-1 10μM
DMSO
RSL3 20μM
Fer-1 10μM
KYSE-30
KYSE-150

Figure S4, Supporting Information

Figure S4. qPCR experiments to assess the expression of CD47 and IL1A in ESCC cell lines under conditions of pharmacological ferroptosis modulation. A) Relative mRNA levels of CD47 and IL1A in KYSE-30 and KYSE-150 cells treated with RSL3 (20 µM), Fer-1 (10 µM), or DMSO control for 24 h. Data are presented as mean ± standard error of the mean (SEM). *P* values were calculated by one-way ANOVA, ns, not significant.

Figure S5

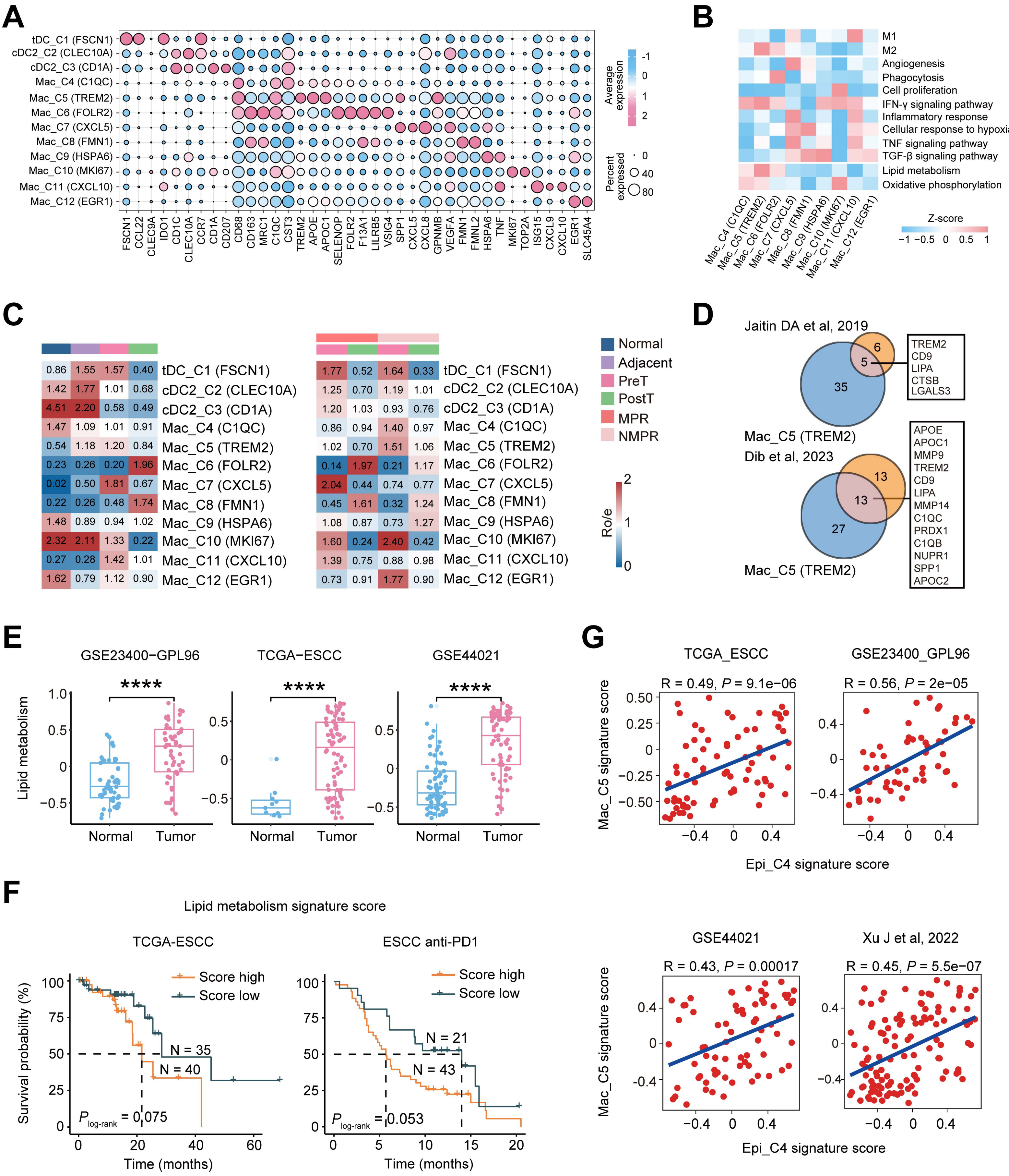
A
B
C
D
E
F
G
Mac_C5 (TREM2)
Jaitin DA et al, 2019
Dib et al, 2023
TREM2
CD9
LIPA
CTSB
LGALS3
APOE
APOC1
MMP9
MMP14
C1QC
PRDX1
C1QB
NUPR1
SPP1
APOC2
Normal
Adjacent
PreT
PostT
MPR
NMPR
Ro/e
Z-score
M1
M2
Angiogenesis
Phagocytosis
Cell proliferation
IFN-γ signaling pathway
Inflammatory response
TNF signaling pathway
TGF-β signaling pathway
Lipid metabolism
Oxidative phosphorylation
GSE23400−GPL96
TCGA−ESCC
GSE44021
Lipid metabolism signature score
ESCC anti-PD1
Score high
Score low
Survival probability (%)
Time (months)
TCGA_ESCC
GSE23400_GPL96
Xu J et al, 2022
R = 0.49, P = 9.1e−06
R = 0.56, P = 2e−05
R = 0.43, P = 0.00017
R = 0.45, P = 5.5e−07
Mac_C5 signature score
Epi_C4 signature score

Figure S5, Supporting Information

Figure S5. Characterization of myeloid cell clusters. A) Dotplot displaying the expression levels of the selected markers in different myeloid cell subtypes. Dot size reflects the fraction of expressing cells and the colors denote normalized gene expression levels. B) Heatmap displaying expression of 12 curated gene signatures across macrophage cell clusters. C) Tissue distribution of different myeloid cells estimated by Ro/e score. D) Overlap of upregulated genes of LAM in our study and two other independent studies. E) Box plots showing the average levels of lipid metabolism signature in the TCGA bulk RNA-Seq and independent microarray datasets of ESCC samples. Datasets include TCGA-ESCC (N = 87), GSE23400 (N = 106), and GSE44021 (N = 146). *P* values were calculated by two-tailed Wilcoxon rank-sum tests, **** $P < 0.0001$. F) Kaplan–Meier analysis of survival time in ESCC patients from the TCGA-ESCC (left panel) and an ESCC anti-PD1 (right panel) dataset, based on lipid metabolism signature score. G) Correlation between PRDM1+ malignant cells and Mac_C5 in four public ESCC bulk RNA-seq cohorts. Cohorts include TCGA-ESCC (N = 75), GSE23400 (N = 53), GSE38129 (N = 30), and an anti-PD-1 treated cohort (N = 64).

Figure S6

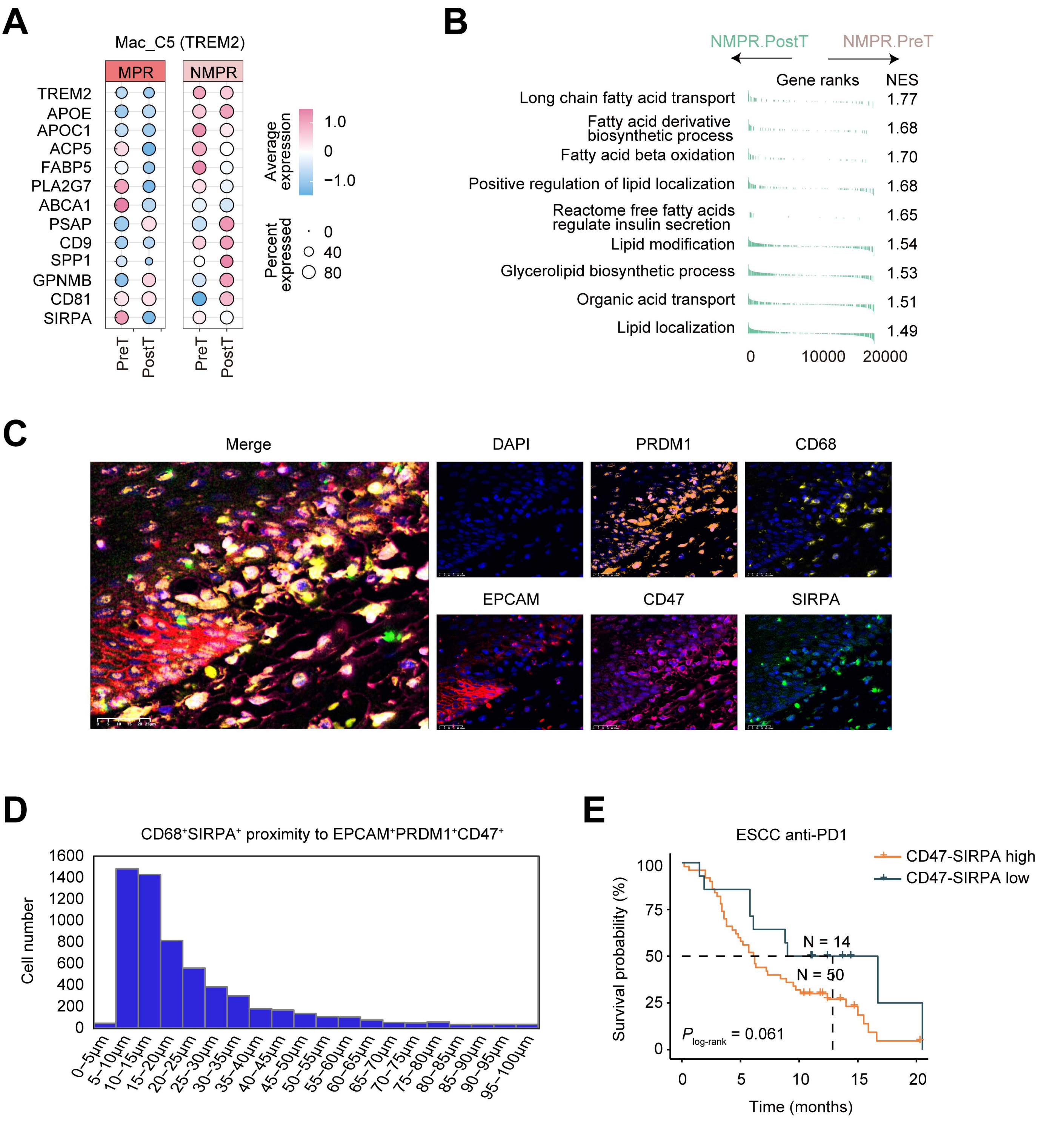

A
Mac_C5 (TREM2)
MPR
NMPR
TREM2
APOE
APOC1
ACP5
FABP5
PLA2G7
ABCA1
PSAP
CD9
SPP1
GPNMB
CD81
SIRPA
PreT
PostT
PreT
PostT
Average expression
1.0
0
−1.0
Percent expressed
0
40
80
B
NMPR.PostT
NMPR.PreT
Gene ranks
NES
Long chain fatty acid transport 1.77
Fatty acid derivative biosynthetic process 1.68
Fatty acid beta oxidation 1.70
Positive regulation of lipid localization 1.68
Reactome free fatty acids regulate insulin secretion 1.65
Lipid modification 1.54
Glycerolipid biosynthetic process 1.53
Organic acid transport 1.51
Lipid localization 1.49
0
10000
20000
C
Merge
DAPI
PRDM1
CD68
EPCAM
CD47
SIRPA
D
CD68⁺SIRPA⁺ proximity to EPCAM⁺PRDM1⁺CD47⁺
Cell number
1600
1400
1200
1000
800
600
400
200
0
0–5μm
5–10μm
10–15μm
15–20μm
20–25μm
25–30μm
30–35μm
35–40μm
40–45μm
45–50μm
50–55μm
55–60μm
60–65μm
65–70μm
70–75μm
75–80μm
80–85μm
85–90μm
90–95μm
95–100μm
E
ESCC anti-PD1
CD47-SIRPA high
CD47-SIRPA low
Survival probability (%)
100
75
50
25
0
N = 14
N = 50
P log-rank = 0.061
0
5
10
15
20
Time (months)

Figure S6, Supporting Information

Figure S6. PRDM1+ malignant cells mediate resistance to nICRT via LAM. A) Dotplots showing the expression levels of lipid metabolism genes in Mac_C5 cells in tumor tissues from MPR and NMPR patients before and after treatment. B) Gene set enrichment analysis showing lipid metabolism-associated pathways were upregulated in Mac_C5 cells after treatment in NMPR patients. The NES are included. C) mIHC staining of EPCAM, PRDM1, CD47, CD68, SIRPA, and DAPI in a NMPR ESCC tumor sample after treatment. Scale bar: 25 µm. D) Quantification using HALO software to analyze the distribution of CD68+SIRPA+ macrophage cells surrounding EPCAM+PRDM1+CD47+ cancer cells in mIHC-stained patient whole-slide images. E) Kaplan–Meier analysis of survival time in patients from an ESCC anti-PD1 dataset, based on the interactions score between Epi_C4 and Mac_C5.

Figure S7

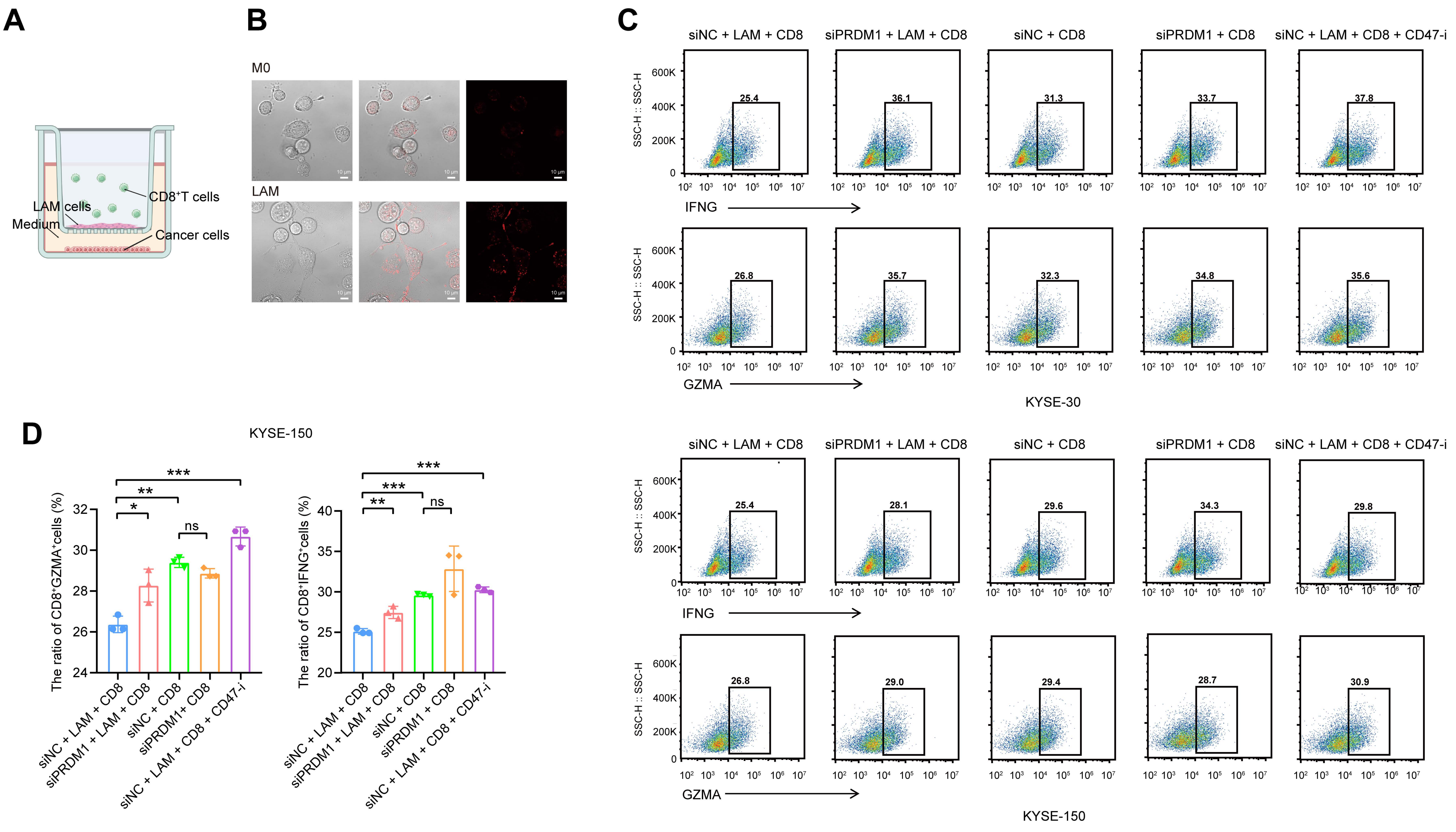

A
B
C
D
CD8⁺T cells
LAM cells
Medium
Cancer cells
M0
LAM
10 μm
siNC + LAM + CD8
siPRDM1 + LAM + CD8
siNC + CD8
siPRDM1 + CD8
siNC + LAM + CD8 + CD47-i
SSC-H :: SSC-H
IFNG
GZMA
25.4
36.1
31.3
33.7
37.8
26.8
35.7
32.3
34.8
35.6
KYSE-30
25.4
28.1
29.6
34.3
29.8
26.8
29.0
29.4
28.7
30.9
KYSE-150
The ratio of CD8⁺GZMA⁺cells (%)
The ratio of CD8⁺IFNG⁺cells (%)
ns

Figure S7, Supporting Information

Figure S7. Results of the co-culture experiments of cancer cells, CD8+ T cells, and LAMs. A) Schematic diagram of the coculture system with cancer cells, CD8+ T cells, and LAMs. B) Nile red staining shows the induction of LAMs, Scale bar: 10μm. C) Flow cytometry gating strategy for identification of IFNG+CD8+ T and GZMA+CD8+ T cell populations. D) Flow cytometry quantification of the proportion of GZMA+CD8+ T cells (left panel) and IFNG+CD8+ T cells (right panel). Data are presented as mean ± SEM, P values were calculated by one-way ANOVA, * $P < 0.05$, ** $P < 0.01$, *** $P < 0.001$; ns, not significant.

Figure S8

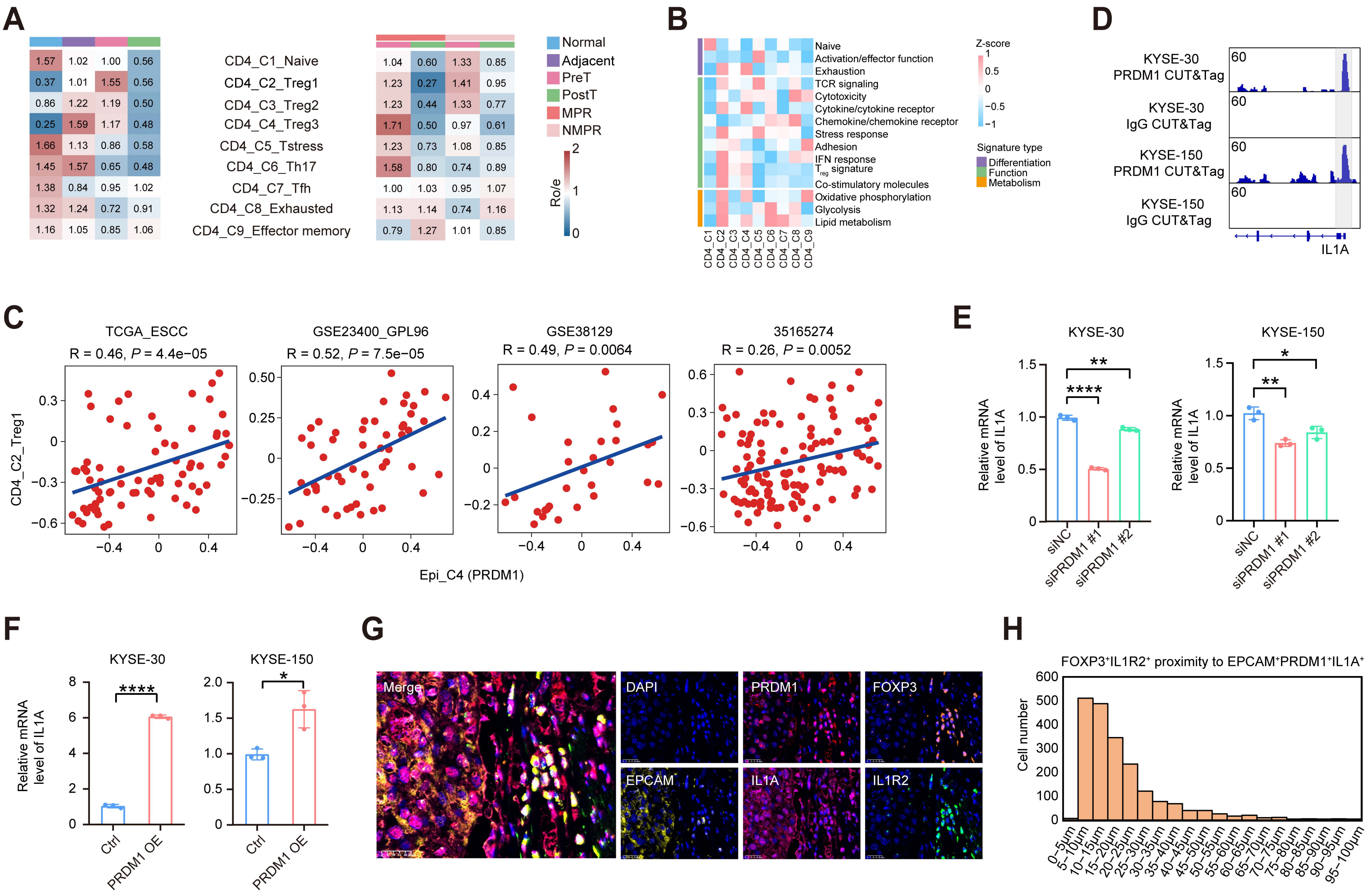

A
CD4_C1_Naive
CD4_C2_Treg1
CD4_C3_Treg2
CD4_C4_Treg3
CD4_C5_Tstress
CD4_C6_Th17
CD4_C7_Tfh
CD4_C8_Exhausted
CD4_C9_Effector memory
Normal
Adjacent
PreT
PostT
MPR
NMPR
Ro/e
B
Naive
Activation/effector function
Exhaustion
TCR signaling
Cytotoxicity
Cytokine/cytokine receptor
Chemokine/chemokine receptor
Stress response
Adhesion
IFN response
Treg signature
Co-stimulatory molecules
Oxidative phosphorylation
Glycolysis
Lipid metabolism
Z-score
Signature type
Differentiation
Function
Metabolism
D
KYSE-30 PRDM1 CUT&Tag
KYSE-30 IgG CUT&Tag
KYSE-150 PRDM1 CUT&Tag
KYSE-150 IgG CUT&Tag
IL1A
C
TCGA_ESCC
R = 0.46, P = 4.4e−05
GSE23400_GPL96
R = 0.52, P = 7.5e−05
GSE38129
R = 0.49, P = 0.0064
35165274
R = 0.26, P = 0.0052
CD4_C2_Treg1
Epi_C4 (PRDM1)
E
KYSE-30
KYSE-150
Relative mRNA level of IL1A
siNC
siPRDM1 #1
siPRDM1 #2
F
KYSE-30
KYSE-150
Relative mRNA level of IL1A
Ctrl
PRDM1 OE
G
Merge
DAPI
PRDM1
FOXP3
EPCAM
IL1A
IL1R2
H
FOXP3+IL1R2+ proximity to EPCAM+PRDM1+IL1A+
Cell number

Figure S8, Supporting Information

Figure S8. CD4 cells profiling in ESCC. A) Tissue distribution of different CD4 cells estimated by Ro/e score. B) Heatmap displaying expression of 15 curated gene signatures across CD4 cell clusters. C) Correlation between PRDM1+ malignant cells and CD4_C2_Treg1 in four public ESCC bulk RNA-seq cohorts. Cohorts include TCGA-ESCC (N = 75), GSE23400 (N = 53), GSE38129 (N = 30), and an anti-PD-1 treated cohort (N = 64). D) Genome browser tracks of PRDM1 CUT&Tag-seq at the IL1A genomic locus in ESCC cells. E) Effects of *PRDM1* depletion (siPRDM1) on *IL1A* expression in ESCC cells. F) Effects of *PRDM1* overexpression on *IL1A* expression in ESCC cells. G) mIHC staining of EPCAM, PRDM1, IL1A, FOXP3, IL1R2, and DAPI in a NMPR ESCC tumor sample after treatment. Scale bars: 25 µm. H) The HALO software was used to quantify the distribution of FOXP3+IL1R2+ Treg cells surrounding EPCAM+PRDM1+IL1A+ cancer cells in mIHC-stained images. Data are presented as mean ± SEM, P values were calculated by unpaired one-way ANOVA (E) or two-tailed Student's t-test (F), * $P < 0.05$, ** $P < 0.01$, **** $P < 0.0001$.

Figure S9

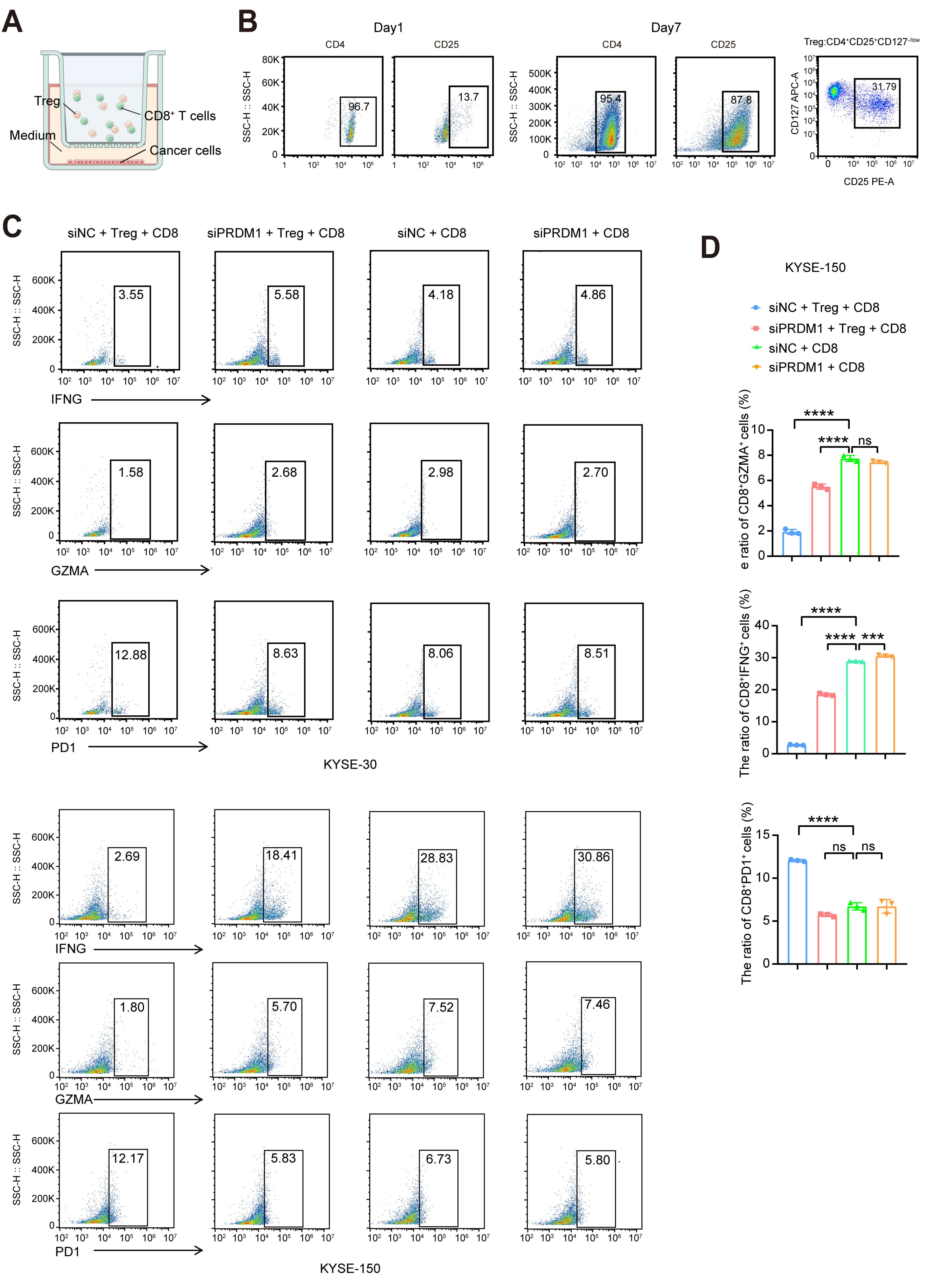

A
Treg
CD8⁺ T cells
Medium
Cancer cells
B
Day1
Day7
CD4
CD25
SSC-H :: SSC-H
96.7
13.7
95.4
87.8
Treg:CD4⁺CD25⁺CD127⁻/low
CD127 APC-A
CD25 PE-A
31.79
C
siNC + Treg + CD8
siPRDM1 + Treg + CD8
siNC + CD8
siPRDM1 + CD8
IFNG
GZMA
PD1
3.55
5.58
4.18
4.86
1.58
2.68
2.98
2.70
12.88
8.63
8.06
8.51
KYSE-30
2.69
18.41
28.83
30.86
1.80
5.70
7.52
7.46
12.17
5.83
6.73
5.80
KYSE-150
D
KYSE-150
siNC + Treg + CD8
siPRDM1 + Treg + CD8
siNC + CD8
siPRDM1 + CD8
e ratio of CD8⁺GZMA⁺ cells (%)
The ratio of CD8⁺IFNG⁺ cells (%)
The ratio of CD8⁺PD1⁺ cells (%)
****
***
ns

Figure S9, Supporting Information

Figure S9. The results of the co-culture experiments of cancer cells, CD8+ T cells, and Tregs. A) Schematic diagram of the coculture system with cancer cells, CD8+ T cells, and Tregs. B) Flow cytometry showing the induction of Tregs cells. C) Flow cytometry quantification of proportions of IFNG+CD8+ T cells (top panel), GZMA+CD8+ T cells (middle panel), and PD1+CD8+ T cells (bottom panel). D) Flow cytometry quantification of proportions of GZMA+CD8+ T cells (top panel), IFNG+CD8+ T cells (middle panel), and PD1+CD8+ T cells (bottom panel). Data are presented as mean ± SEM, *P* values were calculated by one-way ANOVA, *** $P < 0.001$, **** $P < 0.0001$, ns, not significant.

Figure S10

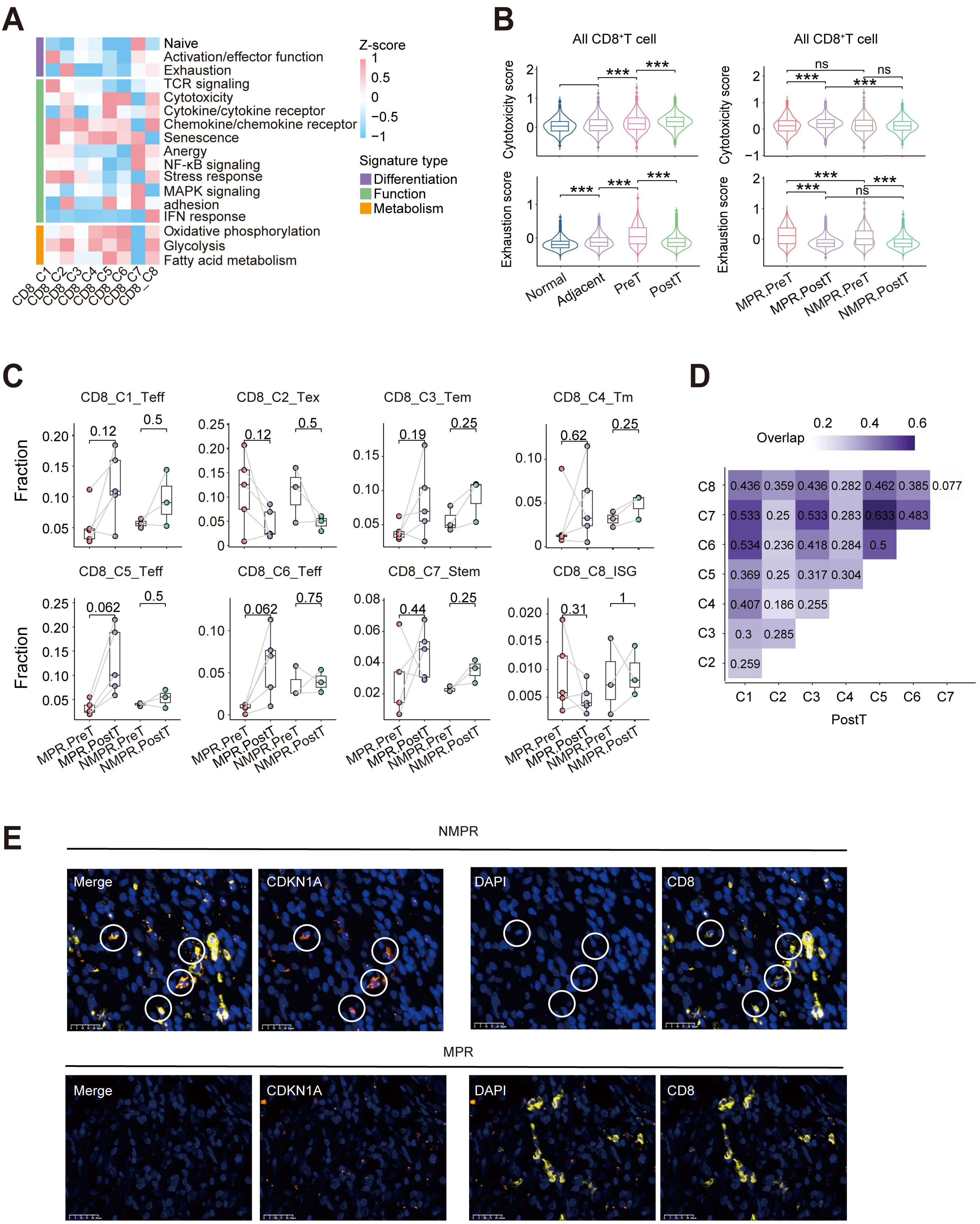
A
Naive
Activation/effector function
Exhaustion
TCR signaling
Cytotoxicity
Cytokine/cytokine receptor
Chemokine/chemokine receptor
Senescence
Anergy
NF-κB signaling
Stress response
MAPK signaling
adhesion
IFN response
Oxidative phosphorylation
Glycolysis
Fatty acid metabolism
CD8_C1 CD8_C2 CD8_C3 CD8_C4 CD8_C5 CD8_C6 CD8_C7 CD8_C8
Z-score
1
0.5
0
−0.5
−1
Signature type
Differentiation
Function
Metabolism
B
All CD8+T cell
Cytotoxicity score
Exhaustion score
***
ns
Normal
Adjacent
PreT
PostT
MPR.PreT
MPR.PostT
NMPR.PreT
NMPR.PostT
C
CD8_C1_Teff
CD8_C2_Tex
CD8_C3_Tem
CD8_C4_Tm
CD8_C5_Teff
CD8_C6_Teff
CD8_C7_Stem
CD8_C8_ISG
Fraction
0.12
0.5
0.19
0.25
0.62
0.062
0.75
0.44
0.31
1
MPR.PreT
MPR.PostT
NMPR.PreT
NMPR.PostT
D
Overlap
0.2
0.4
0.6
C8 0.436 0.359 0.436 0.282 0.462 0.385 0.077
C7 0.533 0.25 0.533 0.283 0.633 0.483
C6 0.534 0.236 0.418 0.284 0.5
C5 0.369 0.25 0.317 0.304
C4 0.407 0.186 0.255
C3 0.3 0.285
C2 0.259
C1 C2 C3 C4 C5 C6 C7
PostT
E
NMPR
MPR
Merge
CDKN1A
DAPI
CD8

Figure S10, Supporting Information

Figure S10. scRNA-seq profiling of CD8 cells in ESCC. A) Heatmap displaying expression of 17 curated gene signatures across CD8+ T cell clusters. B) Violin and box plots of the cytotoxic and exhausted signature scores for all CD8 T cells in different groups. *P* values were calculated by two-tailed Wilcoxon rank-sum tests, *** $P < 0.001$, ns, not significant. C) Boxplots showing the fraction of different CD8 cell subtypes before and after treatment. *P* values were calculated by two-tailed Wilcoxon rank-sum tests D) The heatmap illustrating the clonal overlap among CD8 subtypes after treatment. E) mIHC staining of CD8, CDKN1A, and DAPI in MPR and NMPR ESCC tumor samples. Scale bars: 25 µm.

Figure S11

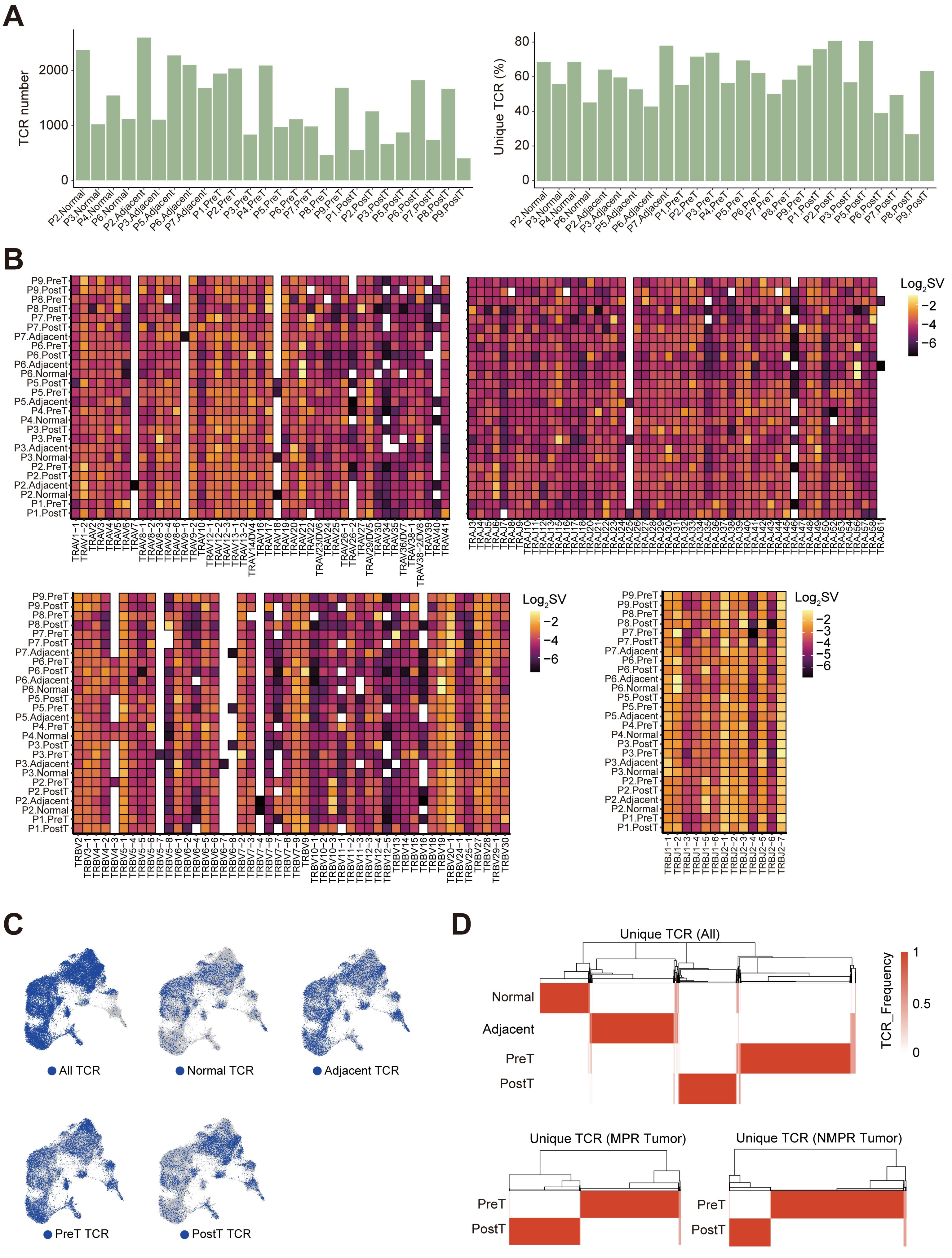

A
TCR number
Unique TCR (%)
B
Log2SV
C
All TCR
Normal TCR
Adjacent TCR
PreT TCR
PostT TCR
D
Unique TCR (All)
Normal
Adjacent
PreT
PostT
TCR_Frequency
Unique TCR (MPR Tumor)
Unique TCR (NMPR Tumor)

Figure S11, Supporting Information

Figure S11. VJ gene usage and pairing and TCR diversity in ESCC patients. A) Bar chart showing the number of TCRs and percent of specific TCRs in each sample. B) Heatmap showing the scaled value of each TCRβ V and J gene segment present in the total T cells from each sample, normalized per column. The gene segments are positioned according to their genomic location. C) UMAP visualization showing different tissue-derived T cells based on their TCR sequences. D) Heatmap showing the frequency of each TCR in the four tissue types. All TCRs in the expanded T cells from tumors are presented. Columns represent the different clonotypes, while the rows represent the tissue types. The color key indicates the frequency of each TCR in each tissue.

Figure S12

A

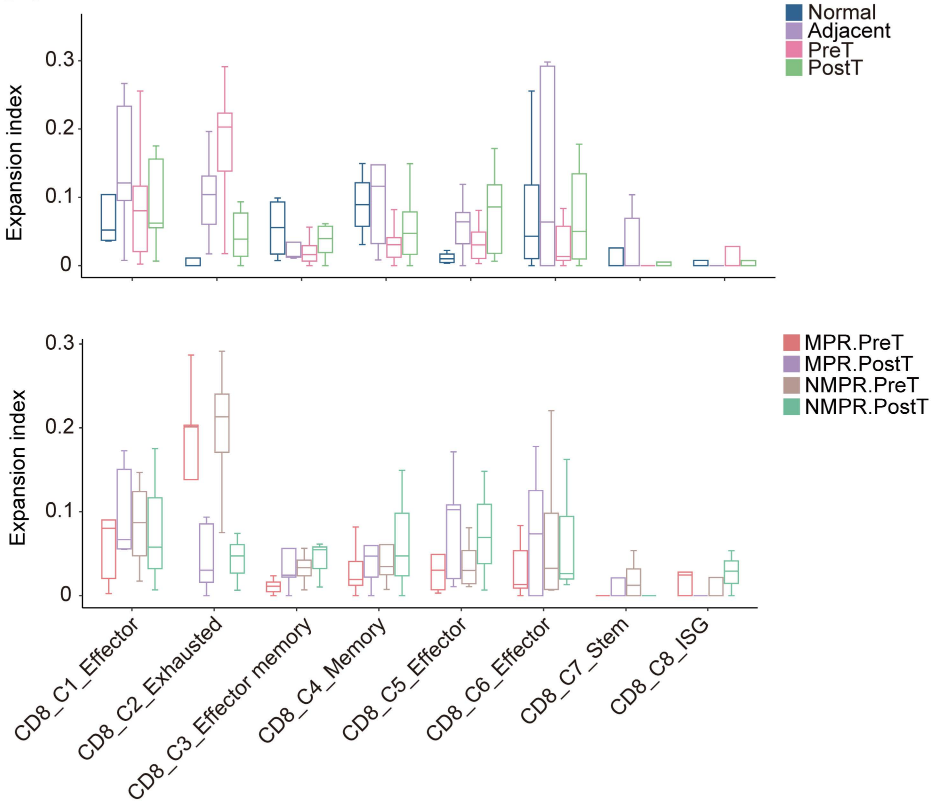

B

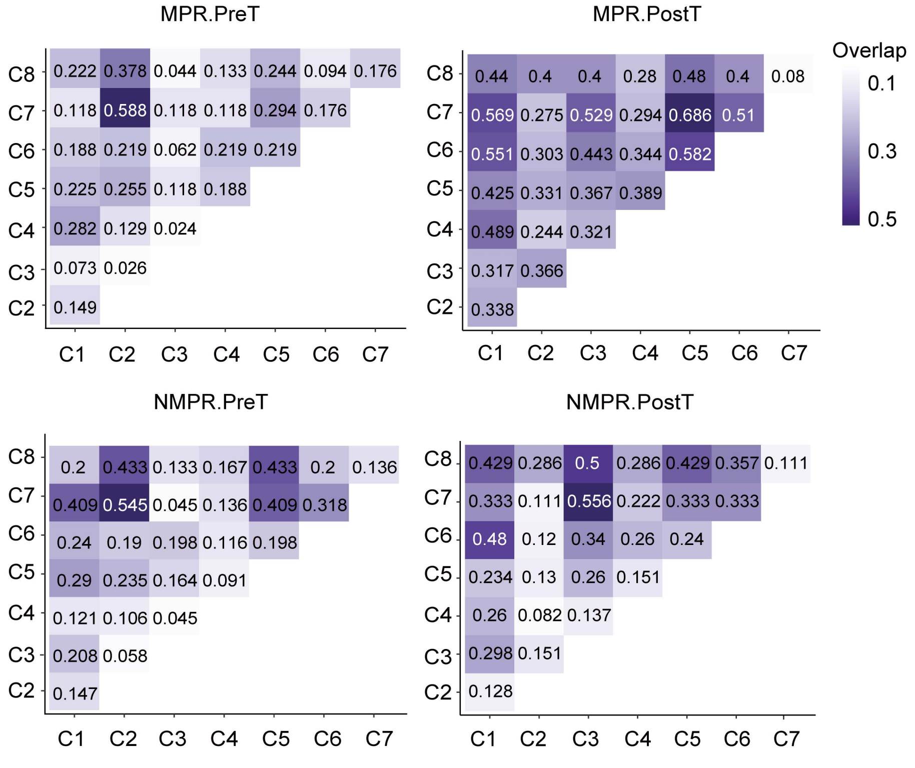

C

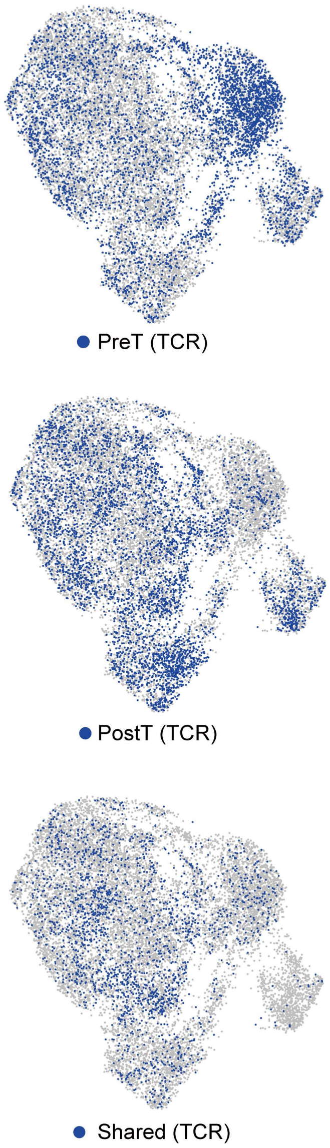

Figure S12, Supporting Information

Figure S12. The expansion state and lineage transition of CD8+ T cells. A) Boxplots of the expansion index for CD8 cell subtypes across different groups. B) The heatmap illustrating the clonal overlap among CD8 subtypes under different groups. C) UMAP showing the pre-tumor TCRs, post-tumor TCRs, and Shared TCRs, according to their TCR sequence.

Figure S13

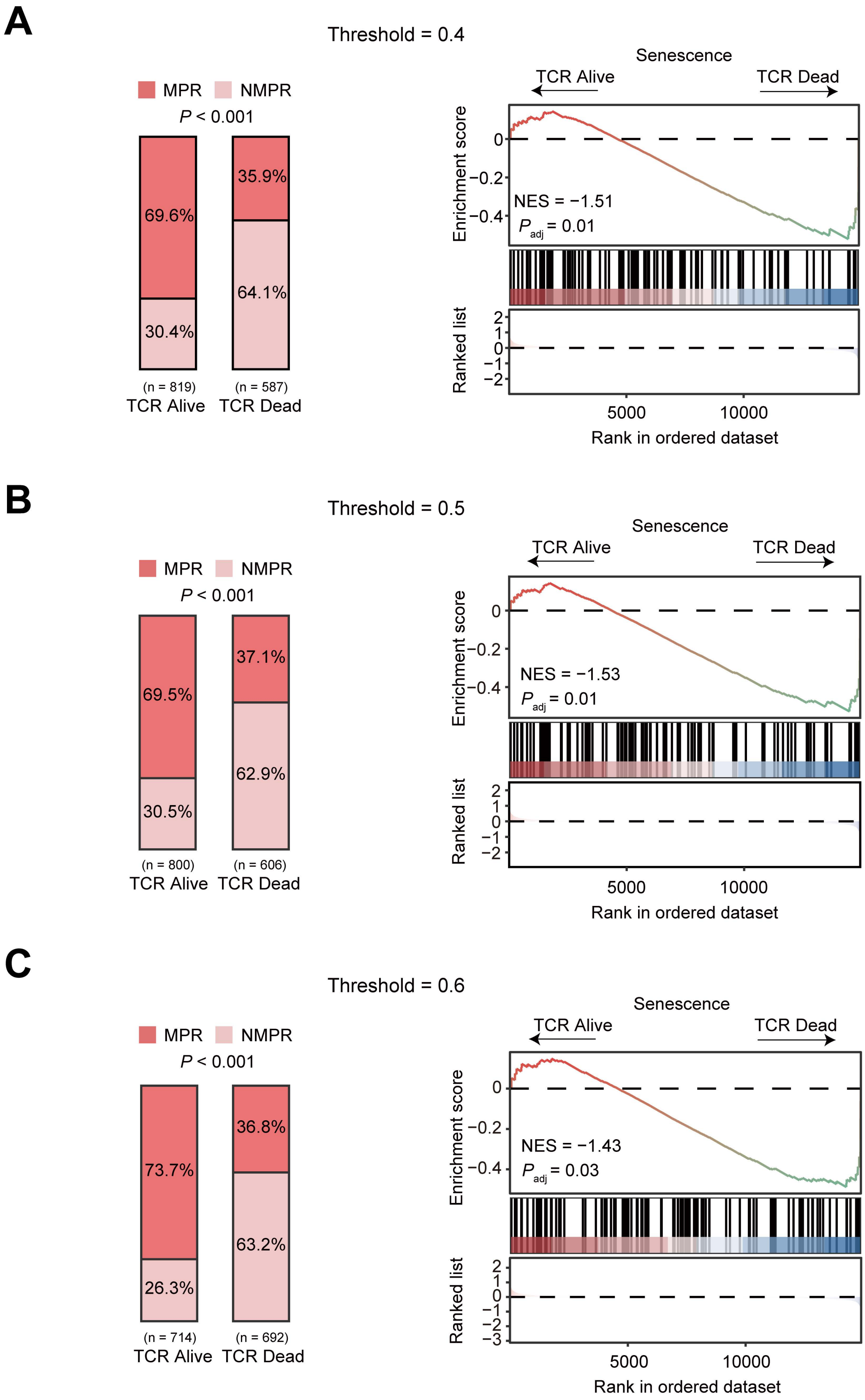
A
Threshold = 0.4
MPR NMPR
P < 0.001
69.6%
30.4%
35.9%
64.1%
(n = 819)
TCR Alive
(n = 587)
TCR Dead
Senescence
TCR Alive
TCR Dead
Enrichment score
0
−0.2
−0.4
NES = −1.51
P adj = 0.01
Ranked list
2
1
0
−1
−2
5000
10000
Rank in ordered dataset
B
Threshold = 0.5
MPR NMPR
P < 0.001
69.5%
30.5%
37.1%
62.9%
(n = 800)
TCR Alive
(n = 606)
TCR Dead
Senescence
TCR Alive
TCR Dead
Enrichment score
0
−0.2
−0.4
NES = −1.53
P adj = 0.01
Ranked list
2
1
0
−1
−2
5000
10000
Rank in ordered dataset
C
Threshold = 0.6
MPR NMPR
P < 0.001
73.7%
26.3%
36.8%
63.2%
(n = 714)
TCR Alive
(n = 692)
TCR Dead
Senescence
TCR Alive
TCR Dead
Enrichment score
0
−0.2
−0.4
NES = −1.43
P adj = 0.03
Ranked list
2
1
0
−1
−2
−3
5000
10000
Rank in ordered dataset

Figure S13, Supporting Information

Figure S13. TCR_Dead cells display a strong senescent phenotype. A-C) Clonally shared CD8 T cells were stratified into two distinct states based on different thresholds (cell number after nICRT treatment/total cell number = 0.4, 0.5, and 0.6). Left panels depict the correlation of each state with treatment efficacy. Right panels from gene set enrichment analysis consistently identify the senescence pathway as significantly upregulated in the TCR_Dead population.
