# Supplementary material for: PRDM1+ Malignant Cells Mediate an Immunosuppressive Landscape and Resistance to Neoadjuvant Chemoradiotherapy and Immunotherapy in Esophageal Squamous Cell Carcinoma
Source: Adv Sci (Weinh). 2026 Jan 20;13(17):e15207. doi: 10.1002/advs.202515207 (PMC13042517; doi:10.1002/advs.202515207)
Supplement: Supplementary file 2 — Supporting File 2: advs73884‐sup‐0002‐Tables.docx. [file ADVS-13-e15207-s001.docx]

**Table S1. Demographic and disease characteristics of the participants at baseline (Intention-to-Treat Population) (N = 22).**

| Characteristic | No. (%) |
| --- | --- |
| Age (years), median (range) | 64 (47–75) |
| ≥ 65 years -No. (%) | 11 (50.0%) |
| Male sex, No. (%) | 17 (77.3) |
| Smoking history, No. (%) | 11 (50.0) |
| Drinking history, No. (%) | 13 (59.1) |
| Tumor location, No. (%) |  |
| Proximal third | 2 (9.1) |
| Middle third | 8 (36.4) |
| Distal third | 12 (54.5) |
| Clinical T stage, No. (%) |  |
| cT2 | 3 (13.6) |
| cT3 | 17 (77.3) |
| cT4a | 2 (9.1) |
| Clinical N stage, No. (%) |  |
| N0 | 2 (9.1) |
| N1 | 11 (50.0) |
| N2 | 9 (40.9) |
| Clinical stage, No. (%) |  |
| II | 4 (18.2) |
| III | 16 (72.7) |
| IVA | 2 (9.1) |
| Performance status (ECOG), No. (%) |  |
| 0 | 19 (86.4) |
| 1 | 3(13.6) |

ECOG, Eastern Cooperative Oncology Group.

**Table S2. Baseline and clinical information of ESCC patients in the present study.**

| **Patient ID** | **Gender** | **Age** | | **Smoker^a)^** | **Drinker^a^** | **Anatomic region** | **Tumor stage^b)^** | **nICRT response** | **Local recurrence** | **Metastasis** | **Sample type** |
| --- | --- | --- | --- | --- | --- | --- | --- | --- | --- | --- | --- |
| Pt1 | M | | 65 | No | Yes | Lower | III | NMPR | No | No | PreT/PostT |
| Pt2 | M | | 74 | Yes | Yes | Middle | III | NMPR | No | Yes | Normal/Adjacent/  PreT/PostT |
| Pt3 | M | | 55 | No | No | Lower | III | NMPR | No | Yes | Normal/Adjacent/  PreT/PostT |
| Pt4 | M | | 53 | Yes | Yes | Lower | IVA | NMPR | No | Yes | Normal/PreT |
| Pt5 | F | | 68 | No | No | Lower | III | MPR | No | No | Adjacent/  PreT/PostT |
| Pt6 | M | | 73 | Yes | No | Lower | III | MPR | No | No | Normal/Adjacent/  PreT/PostT |
| Pt7 | M | | 55 | Yes | Yes | Lower | III | MPR | No | No | Adjacent/  PreT/PostT |
| Pt8 | M | | 60 | Yes | Yes | Lower | III | MPR | No | No | PreT/PostT |
| Pt9 | F | | 71 | No | No | Lower | III | MPR | No | No | PreT/PostT |

^a)^ Individuals who smoked an average of <1 cigarette/day and for <1 year in their lifetime were defined as nonsmokers; otherwise, they were defined as smokers. Individuals were classified as drinkers if they drank at least twice a week and continuously for at least 1 year during their lifetime; otherwise, they were defined as nondrinkers.

^b)^ Tumor staging was reviewed by at least 3 pathologists and defined according to the American Joint Committee on Cancer (AJCC) 7th edition.

**Table S3. Sequences of primers and siRNAs used in this study.**

| **qRT-PCR** | **Primer sequence (5' → 3')** |
| --- | --- |
| PRDM1-Forward | CAGCGACGAAGCCATGAATC |
| PRDM1-Reverse | AGCCCTTGTTGCAAGTCTGA |
| MFSD12-Forward | GTGTACGGCTCCATGAGCTT |
| MFSD12-Reverse | AGCAGGAGGCTACAGAGACA |
| CTSB-Forward | CTGACCGGATCTGCATCCAC |
| CTSB-Reverse | CTTCAGCAGGATAGCCACCA |
| CD47-Forward | TGAGTCTCTGTATTGCGGCG |
| CD47-Reverse | GGGTTCCTCTACAGCTTTCCT |
| IL1A-Forward | GTAGCAACCAACGGGAAGGT |
| IL1A-Reverse  GAPDH-Forward  GAPDH-Reverse | AAGGTGCTGACCTAGGCTTG  CGCTGAGTACGTCGTGGAGTC  GCTGATGATCTTGAGGCTGTTGTC |
| **siRNAs** | **Sequence (5' → 3')** |
| siNC | UUCUCCGAACGUGUCACGUTT |
| siPRDM1 #1 | GGAAAGGACCUCUACCGUUTT |
| siPRDM1 #2 | GATCTGACCCGAATCAATG |
